# Molecular determinants of cross-reactivity and potency by VH3-33 antibodies against the *Plasmodium falciparum* circumsporozoite protein

**DOI:** 10.1016/j.celrep.2023.113330

**Published:** 2023-10-28

**Authors:** Elaine Thai, Rajagopal Murugan, Špela Binter, Clare Burn Aschner, Katherine Prieto, Audrey Kassardjian, Anna S. Obraztsova, Ryu Won Kang, Yevel Flores-Garcia, Shamika Mathis-Torres, Kan Li, Gillian Q. Horn, Richard H.C. Huntwork, Judith M. Bolscher, Marloes H.C. de Bruijni, Robert Sauerwein, S. Moses Dennison, Georgia D. Tomaras, Fidel Zavala, Paul Kellam, Hedda Wardemann, Jean-Philippe Julien

**Affiliations:** 1 Program in Molecular Medicine, The Hospital for Sick Children Research Institute, Toronto, ON M5G 0A4, Canada; 2 Department of Biochemistry, University of Toronto, Toronto, ON M5S 1A8, Canada; 3 B Cell Immunology, German Cancer Research Center (DKFZ), 69120 Heidelberg, Germany; 4 Kymab Ltd./Sanofi, The Bennet Building (B930), Babraham Research Campus, Cambridge CB22 3AT, UK; 5 RQ Biotechnology Limited, 7th Floor Lynton House, 7–12 Tavistock Square, London WC1H 9LT, UK; 6 Department of Immunology, University of Toronto, Toronto, ON M5S 1A8, Canada; 7 Biosciences Faculty, University of Heidelberg, 69117 Heidelberg, Germany; 8 Department of Molecular Microbiology and Immunology, Malaria Research Institute, Johns Hopkins Bloomberg School of Public Health, Baltimore, MD 21205, USA; 9 Departments of Surgery, Integrative Immunobiology, Molecular Genetics, and Microbiology, Center for Human Systems Immunology, Duke University, Durham, NC 27710, USA; 10 TropIQ Health Sciences, 6534 AT Nijmegen, the Netherlands; 11 Department of Infectious Diseases, Faculty of Medicine, Imperial College London, London SW7 2BX, UK

**Keywords:** CSP, circumsporozoite protein, malaria, antibody, *Plasmodium falciparum*, cross-reactivity, repeat motifs, Fab structure, VH3-33 antibodies

## Abstract

*IGHV3-33*-encoded antibodies are prevalent in the human humoral response against the *Plasmodium falciparum* circumsporozoite protein (PfCSP). Among VH3-33 antibodies, cross-reactivity between PfCSP major repeat (NANP), minor (NVDP), and junctional (NPDP) motifs is associated with high affinity and potent parasite inhibition. However, the molecular basis of antibody cross-reactivity and the relationship with efficacy remain unresolved. Here, we perform an extensive structure-function characterization of 12 VH3-33 anti-PfCSP monoclonal antibodies (mAbs) with varying degrees of cross-reactivity induced by immunization of mice expressing a human immunoglobulin gene repertoire. We identify residues in the antibody paratope that mediate cross-reactive binding and delineate four distinct epitope conformations induced by antibody binding, with one consistently associated with high protective efficacy and another that confers comparably potent inhibition of parasite liver invasion. Our data show a link between molecular features of cross-reactive VH3-33 mAb binding to PfCSP and mAb potency, relevant for the development of antibody-based interventions against malaria.

## Highlights

• X-ray crystal structures of 12 VH3-33 mAbs bound to PfCSP peptides reveal four binding modes
• Specific HCDR features influence the induced epitope conformation and mAb cross-reactivity
• The 1210-like binding mode mediates strong cross-reactivity and inhibitory efficacy
• A unique binding conformation confers potent inhibition despite low cross-reactivity

## Graphical abstract

**Figure:**
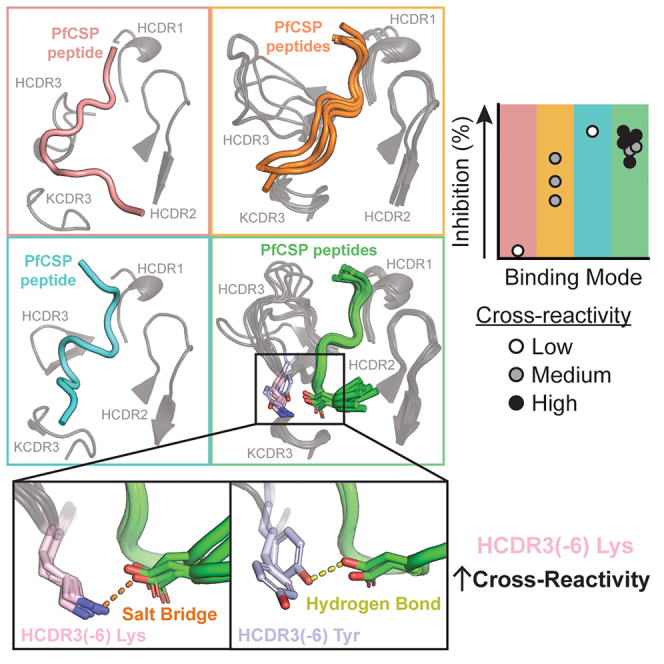

## Teaser

Thai et al. provide a comprehensive structure-function assessment of 12 VH3-33 mAbs with varying degrees of cross-reactivity for the PfCSP repeat motifs. High-affinity cross-reactive binding and potent inhibitory function are consistently associated with one PfCSP recognition mode, for which specific HCDR features forming the underlying molecular basis are identified.

## Introduction

*Plasmodium* parasites are the causative agents of malaria, with *Plasmodium falciparum* (Pf) being the deadliest to humans. Global malaria elimination efforts continue to be threatened by increasing resistance of the transmitting *Anopheles* mosquitoes to insecticides and of Pf to antimalarial medicines, resulting in consistent reports of ∼240 million cases every year for the past two decades.^1^ Thus, a potent anti-infection biomedical intervention is sorely needed. While most antimalarial medicines target the parasite’s liver and/or blood stages,^2^ effective antibody-mediated neutralization of the pre-erythrocytic sporozoite stage presents an opportunity to prevent infection, thereby providing protection against the disease.^3^

As the most abundant surface protein expressed by Pf sporozoites and with essential roles in parasite development and invasion, Pf circumsporozoite protein (PfCSP) is a major target for biomedical interventions.^4^^,^^5^^,^^6^ PfCSP is composed of three domains: an N-terminal domain that undergoes proteolytic cleavage prior to hepatocyte invasion; a polymorphic, T cell epitope-containing C-terminal domain; and a conserved central region largely composed of consecutive major NANP repeat motifs.^7^^,^^8^^,^^9^^,^^10^ The junction that links the N-terminal domain to the central NANP repeats contains a singular junctional NPDP motif, followed by three minor NVDP repeats, each interspersed with an NANP motif.^7^

While monoclonal antibodies (mAbs) against the N- and C-terminal domains have exhibited poor parasite inhibition, those specific for each of the tetrapeptide motifs (e.g., mAb 317 [NANP], mAb L9 [NVDP], and mAb CIS43 [NPDP]) have demonstrated sporozoite neutralization in animal models and phase I clinical trials where protective efficacy was evaluated against controlled human malaria infection.^11^^,^^12^^,^^13^^,^^14^^,^^15^^,^^16^^,^^17^^,^^18^^,^^19^^,^^20^ Because of the high sequence similarity between the PfCSP repeat motifs, mAbs with high affinity for one motif tend to cross-react with the other repeat motifs, albeit with reduced affinity. In this way, cross-reactivity between the junctional, minor, and major PfCSP repeat motifs is associated with increased affinity and potent parasite inhibition.^21^ Only a small subset of mAbs have been observed to cross-bind indiscriminately between the three distinct repeat motifs (e.g., mAbs 4493 and 2541).^21^ Consequently, it remains unclear how the molecular features underlying cross-reactive antibody binding are associated with parasite inhibitory function.

Extensive structural characterization of anti-PfCSP repeat mAbs has resulted in the elucidation of a wide range of PfCSP recognition modes because different inhibitory mAbs induce different binding conformations for otherwise largely disordered repeat motifs.^15^^,^^16^^,^^17^^,^^21^^,^^22^^,^^23^^,^^24^^,^^25^^,^^26^^,^^27^^,^^28^^,^^29^^,^^30^^,^^31^ This diversity is further amplified by differential heavy-chain gene usage among these mAbs, including *IGHV3-30* (mAb 317),^15^ *IGHV1-3* (mAb CIS43),^17^ *IGHV3-49* (mAb 4493),^21^ and *IGHV3-33* (mAb L9).^18^ As a result of such broad diversity, although distinct antibody-bound epitope conformations have recently been linked to varying levels of potency,^27^ the effects of binding mode on mAb inhibitory efficacy are still ambiguous. Therefore, to facilitate an investigation of the complex relationships between antibody binding conformation, cross-reactivity, and potency for a common genetic background, we focused specifically on *IGHV3-33*-encoded antibodies because they have been reported to be of high prevalence in the anti-PfCSP humoral response.^15^^,^^17^^,^^21^^,^^32^^,^^33^

Here, to obtain high-affinity cross-reactive VH3-33 mAbs for molecular characterization, we immunized mice carrying human immunoglobulin (Ig) loci (Kymouse platform^34^) with nanocage-based immunogens presenting the junctional, minor, and major PfCSP repeat motifs.^35^ We selected 12 VH3-33 mAbs of varying degrees of cross-reactivity to determine how antibody binding conformation, cross-reactivity, and potency are linked. This collection of mAbs covered a broad range of inhibitory capacities *in vivo* despite demonstrating comparable *in vitro* functionality. In total, 22 antibody fragment (Fab)-antigen complex structures were solved at 1.54- to 2.95-Å resolution to gain insights into whether differences in potency are associated with different features of PfCSP repeat recognition. Our findings provide a comprehensive structure-function relationship for high-affinity, cross-reactive PfCSP antibodies encoded by the predominant *IGHV3-33* gene recruited to the anti-infective humoral response against Pf.

## Results

### High-affinity, cross-reactive VH3-33 antibodies were elicited by nanocage immunogens containing junctional, minor, and major PfCSP repeat motifs

**Figure 1:**
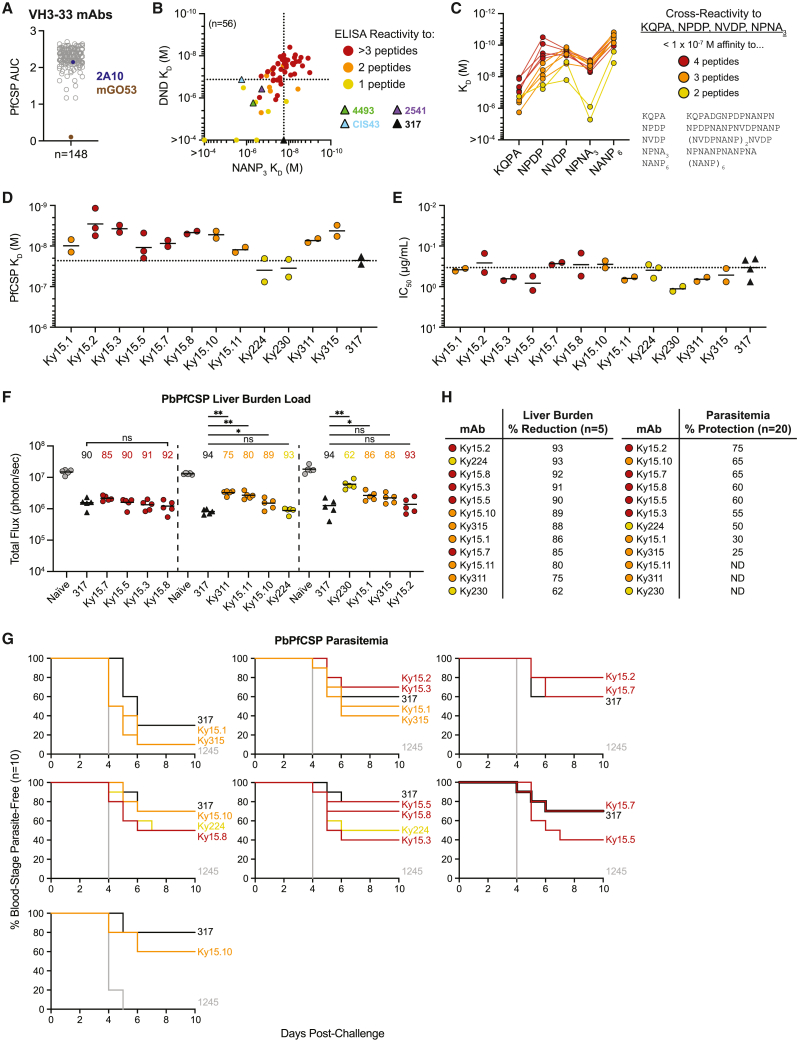
High-affinity cross-reactive VH3-33 mAbs show broad range of parasite-inhibitory capacity (A) Binding of 148 VH3-33 mAbs to PfCSP represented as area under the ELISA binding curve (AUC). mAbs 2A10 (violet) and mGO53 (brown) were used as positive and negative controls, respectively. Symbols represent the mean of three independent experiments. See also Figure S1 and Table S1. (B) DND vs. NANP_3_ affinity of 56 VH3-33 mAbs as measured by SPR. Circular symbols represent a single measurement for each sample mAb and are colored based on cross-reactivity, as detected by ELISA. Triangular symbols represent three independent measurements for previously characterized mAbs and are colored according to the legend on the right. Dotted lines indicate the highest affinity for each peptide observed among the four literature mAbs. (C) SPR binding profiles of 12 selected mAbs to the indicated peptides. Symbols represent the mean of three independent measurements. (D) Biolayer interferometry (BLI) binding of mAbs to recombinant PfCSP. (E) Half-maximal inhibitory capacity (IC_50_) of mAbs against Pf sporozoites measured by the *in vitro* traversal assay. (D and E) Symbols represent independent experiments, and black lines indicate geometric mean. Dotted lines indicate mAb 317 mean PfCSP K_D_ and IC_50_. (F) Total flux reflecting PbPfCSP liver load in three independent *in vivo* liver burden assays following passive transfer with 100 μg of the indicated mAb. Symbols represent individual mice and dashed lines separate independent experiments. Percent reduction relative to naive mice is shown above the corresponding sample groups. Black lines indicate arithmetic mean. Naive mice are used as negative controls and shown as gray symbols. Statistical significance was determined by two-tailed Mann-Whitney test (n = 5): ns, not significant; ^∗^p < 0.05, ^∗∗^p < 0.01. (G) PbPfCSP infection curves following passive transfer with 150 μg of the indicated mAb prior to challenge from ∼5 infected mosquito bites. Each mAb was evaluated in two replicate experiments (n = 10 per experiment), and each plot shows an independent experiment. All sample mAbs were found to be significantly different than negative control mAb 1245 (gray) and not significantly different than mAb 317 using the Mantel-Cox log rank test (n = 20) with Bonferroni correction applied. (D–G) mAb 317 is used as a positive control and is represented by black triangular symbols (D–F) or black curves (G). (H) mAb *in vivo* efficacy summary tables arranged in decreasing order of potency for each assay; ND, not determined. (C–H) mAb symbols and curves are colored based on cross-reactive binding to KQPA, NPDP, NVDP, and NPNA_3_ peptides, as indicated in the legend to the right of (C). See also Figure S2 and Table S2.

To induce cross-reactive antibodies, we designed immunogens presenting the PfCSP strain NF54 junction (KQPADGNPDP[NANPNVDP]_3_), followed by consecutive NANP repeats of varied length (5 [NANP_5_] or 18 [NANP_18_] units) on the surface of *Helicobacter pylori* apoferritin (Ferr)^35^^,^^36^ or *Aquifex aeolicus* lumazine synthase (LS)^37^ nanocages (Figure S1A). Subcutaneous homologous or heterologous prime-boost-boost immunization of mice from the Kymouse platform with these immunogens in the Sigma Adjuvant System (SAS) induced a strong VH3-33 antibody response amongst PfCSP-reactive B cells. From single germinal center B cells and plasma cells isolated from draining lymph nodes and plasma cells isolated from bone marrows of these mice, we generated 148 VH3-33 mAbs that showed strong binding to full-length PfCSP in ELISA (Figures 1A and S1B; Table S1). More than 60% of the mAbs used Vk1-5 kappa light chains (KC), a combination linked previously to high PfCSP affinity,^16^^,^^32^^,^^35^ while the rest had a variety of other kappa and lambda light chains (Figures S1C and S1D). To determine their degree of cross-reactivity to the PfCSP repeats,^21^ mAbs were tested in ELISA for binding to overlapping peptides covering the junction (KQPA, KQPADGNPDPNANP; NPDP, NPDPNANPNVPDNANP; DND, NVDPNANPNVDP; NDN, NANPNVDPNANP) and to a short major repeat peptide (NANP_3_, NANPNANPNANP; Figure S1E). Of all the VH3-33 mAbs, 79 (53%) bound at least three of these peptides and were considered cross-reactive (Figure S1F). The other mAbs showed weak binding (Figure S1G) or preferentially bound one or two peptides (Figure S1H).

To examine a broad range of antibodies in more detail, we selected 56 VH3-33 mAbs to measure their binding affinity to specific peptides by surface plasmon resonance (SPR; Table S1). mAbs were selected from each of the observed binding profiles, with seven different KC genes (Vk1-5, Vk2-24, Vk2-30, Vk3-11, Vk3-15, Vk3D-15, and Vk3-20). DND and NANP_3_ peptides were selected for binding measurements because we have reported previously that high affinity to (N/D)PNANPN(A/V), a core epitope centered around the major repeat, is a feature of protective PfCSP antibodies.^21^ In line with our earlier findings,^21^ binding affinity to NANP_3_ was paralleled by similar affinity for peptide DND, and cross-reactivity was associated with high affinity to both peptides (Figure 1B). In direct comparisons, many of these mAbs exhibited similar or even higher affinities than mAbs 317, CIS43, 2541, and 4493^15^^,^^17^^,^^21^ (Figure 1B). Therefore, by immunizing mice from the Kymouse platform with nanocage immunogens containing the junctional, minor, and major PfCSP repeat motifs, we obtained a large collection of VH3-33 mAbs, including many that demonstrated strong cross-reactivity and high affinity to NANP-centered peptides beyond that of potent mAbs isolated from humans.

### Pf sporozoite inhibition activity of high-affinity, cross-reactive VH3-33 mAbs

We next selected 12 VH3-33 mAbs with varying degrees of cross-reactivity against a large set of PfCSP-derived peptides (KQPA, NPDP, NVDP ((NVDPNANP)_2_NVDP), NPNA_3_ (NPNANPNANPNA), NANP_6_ (NANPNANPNANPNANPNANPNANP)) for further investigation of their *in vitro* functionality and *in vivo* protective efficacy (Figure 1C; Table S2). The selected mAbs exhibited a variety of binding profiles and included two mAbs with low affinity for the NPNA_3_ peptide. 8 of the 12 mAbs were paired with Vk1-5 (Ky15.1, Ky15.2, Ky15.3, Ky15.5, Ky15.7, Ky15.8, Ky15.10, and Ky15.11), whereas the remaining four each used a different KC gene (Vk2-24, Vk2-30, Vk3-11 and Vk3-15; Ky224, Ky230, Ky311, and Ky315, respectively). All 12 mAbs bound recombinant PfCSP with nanomolar affinity (Figure 1D) and effectively inhibited Pf sporozoite traversal *in vitro* with a half-maximal inhibitory capacity (IC_50_) of less than 1.5 μg/mL (Figure 1E), comparable with previously characterized highly potent NANP-specific mAb 317.^15^ Upon passive transfer of C57BL/6 mice with 100 μg of each mAb followed by intravenous challenge with transgenic *Plasmodium berghei* sporozoites expressing PfCSP (PbPfCSP), the 12 VH3-33 mAbs reduced the parasite liver load to varying degrees compared with naive mice that did not receive any antibody (62%–93%; Figures 1F and S2A). To determine whether the mAbs could also protect from the development of blood parasitemia, the nine most potent mAbs that reduced parasite liver burden by at least 85% were each passively transferred into C57BL/6 mice prior to challenge with PbPfCSP sporozoites via mosquito bite. Importantly, to resolve differences in protective capacity among these high-affinity mAbs, a suboptimal dose of 150 μg/mouse was used, resulting in 30%–80% mean protection by mAb 317 across all experiments, with an overall mean of 64% protection (n = 70; Figures 1G and S2B). The nine mAbs of interest resulted in a 25%–75% range in mean protection against parasitemia (n = 20), demonstrating efficacy comparable with mAb 317 (Figures 1G and S2B). Within this subset of high-affinity mAbs (Figures 1C and 1D; Table S2), we noticed that those with stronger cross-reactivity tended to be associated with higher efficacy in liver burden and parasitemia models (Figure 1H). Through simple linear regression, we observed a number of *in vitro* measurements that displayed trends with liver burden (NPDP *k*_*off*_, NVDP *k*_*off*_, NVDP K_D_, PfCSP *k*_*off*_, Pf sporozoite traversal IC_50_) or parasitemia (NANP_3_ *k*_*on*_) inhibition; however, for potent mAbs with 75% or greater liver burden inhibition or 50% or greater parasitemia inhibition, *in vitro* measurements were not absolutely predictive of mAb efficacy in either *in vivo* assay (Figures S2C and S2D). Thus, using this unique set of 12 VH3-33 mAbs that possess a broad range of Pf sporozoite-inhibitory efficacies, we observed that differences in antibody functionality could not easily be explained by single binding affinity parameters alone and set out to explore whether specific molecular features were associated with functionality.

### Common HCDR3 features induce a shared C-terminal core peptide conformation

**Figure 2:**
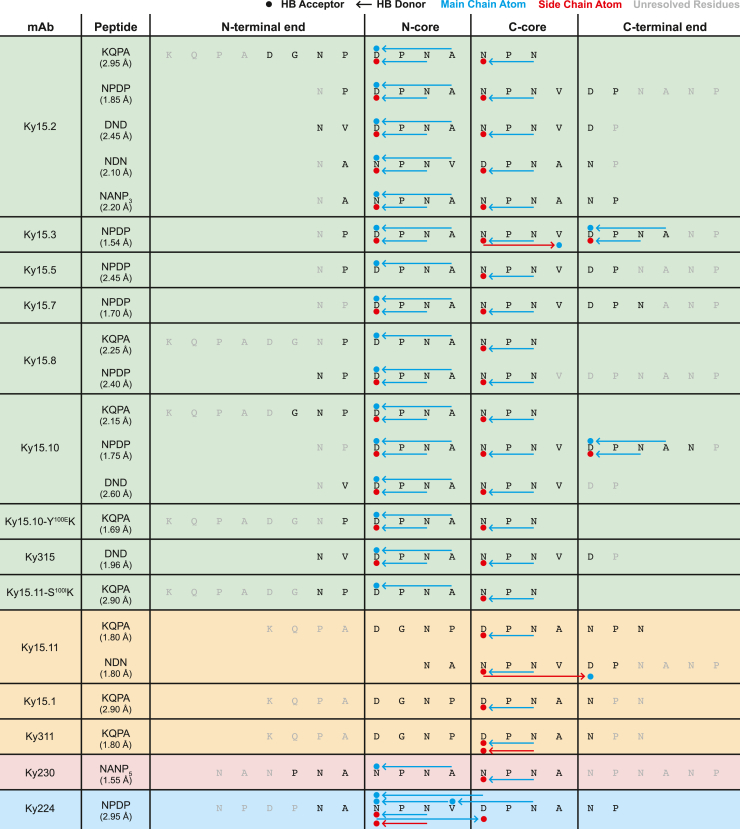
Intrachain hydrogen bonds (HBs) within mAb-bound peptides For each crystallized Fab-peptide complex, the peptide sequence is shown with Fab-bound N-core and C-core motifs positioned as indicated and unresolved residues colored in gray. Filled circles and arrows represent HBs, noting acceptors and donors, respectively, with donor residues at the tail end of the arrow. Main chain donor/acceptor atoms are indicated by blue symbols, and side chain donor/acceptor atoms are depicted as red symbols. HBs mediated solely by main-chain atoms are represented by symbols above the peptide sequence, whereas side-chain-mediated HBs are shown below the sequence. 1210-like structures are shaded with a green background, MGG4-like structures are shaded orange, and Ky230 and Ky224 structures with distinct binding modes are shaded in pink and blue, respectively. The resolution of each Fab-peptide structure is indicated below the corresponding peptide. See also Table S3. X-ray crystallography data collection and refinement data for 1210-like mAbs and variants, related to Figures 2–5, Table S4. X-ray crystallography data collection and refinement data for MGG4-like mAbs and variants, related to Figures 2–4, Table S5. X-ray crystallography data collection and refinement data for Ky230 and Ky224, related to Figures 2 and 7.

**Figure 3:**
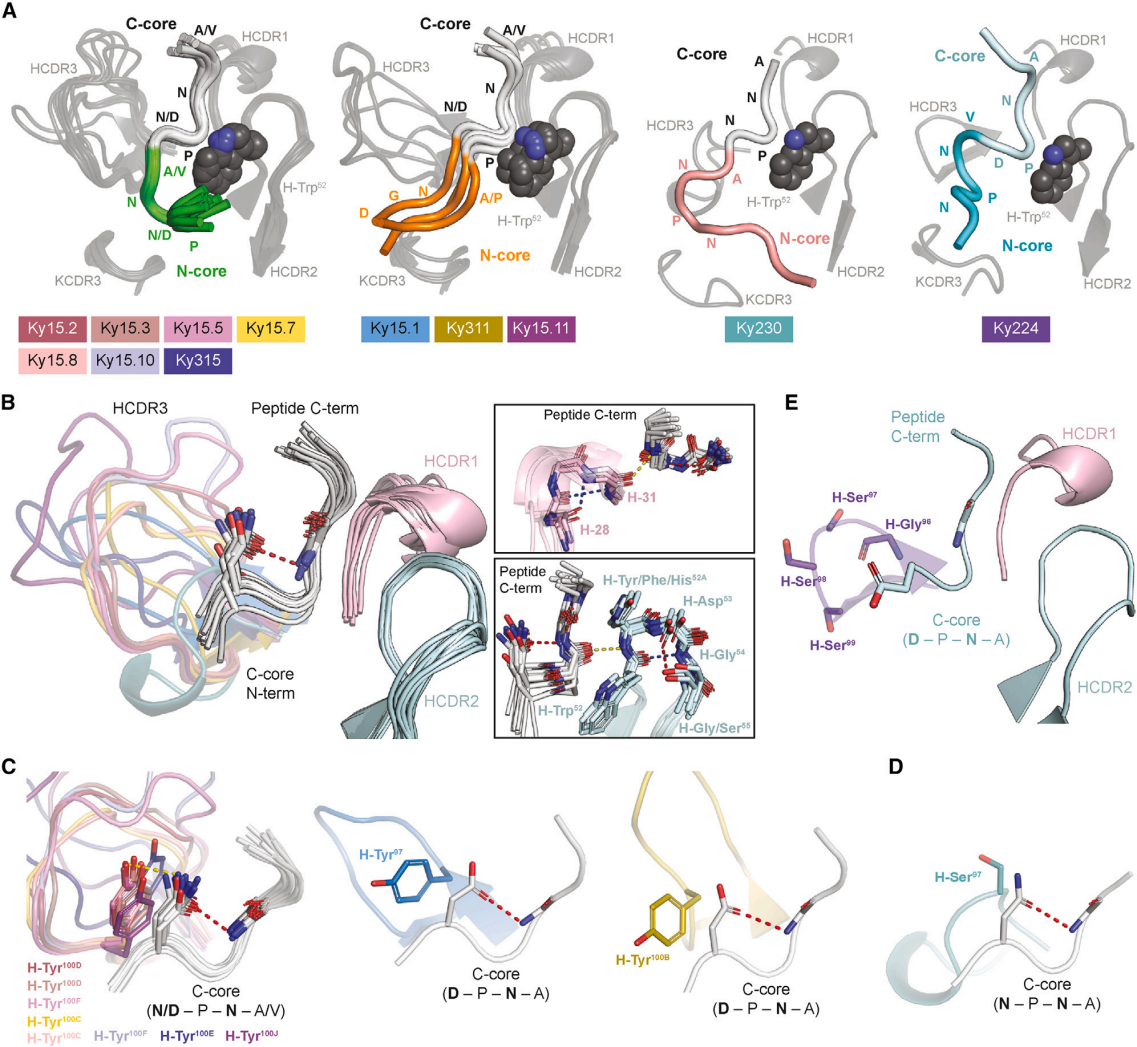
A common C-core conformation is induced by shared HCDR features of VH3-33 mAbs (A) Overview of N- and C-core motifs of different binding conformations. Common C-core conformations are colored in light gray, the unique C-core conformation induced by Ky224 is shown in pale cyan, and N-core motifs are shown in green, orange, salmon, and cyan. Positions of N- and C-core amino acids (aa) are labeled. Structures of mAbs exhibiting the same PfCSP recognition mode are superposed, with HCDRs and KCDR3s in dark gray cartoon representation and VH3-33 GL residue H-Trp^52^ shown as spheres. The legend below indicates which peptide conformation is induced by which mAb. (B) C-core conformation of various peptides (gray) bound independently by 11 different mAbs. HCDR1s and HCDR2s are shown in light pink and pale cyan, respectively. Peptide C-terminal (C-term) and C-core N-terminal (N-term) ends are labeled, and residues facilitating the Asx turn are represented as sticks. Insets on the right highlight features of HCDR1 and HCDR2 that are shared between the 11 mAbs and stabilize the C-core conformation. (C and D) HCDR3 Tyr (C) and Ser (D) residues supporting the C-core Asx turn through stacking interactions and HBs. Interacting residues are indicated in bold text and shown as sticks. Tyr residues in the same structural position and rotameric state are overlaid in a single panel. (B–D) Red and blue dashed lines represent intrachain HBs mediated by side-chain atoms and main-chain atoms, respectively. Yellow dashed lines represent interchain HBs. (E) C-core conformation of the NPDP peptide (pale cyan) bound by Ky224. HCDR1 and 2 are colored as in (B). HCDR3 residues are labeled and shown as sticks. The peptide C-term is labeled, and C-core residues corresponding to those mediating the Asx turn observed in other binding modes are represented as sticks and indicated in bold text. (B–E) HCDR3s are colored by mAb according to the legend in (A). See also Figure S3.

**Figure 4:**
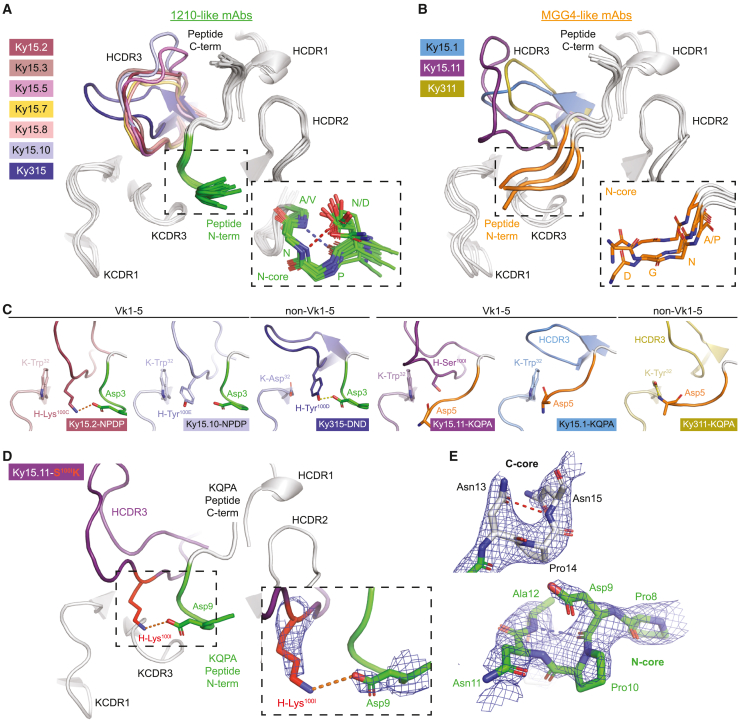
HCDR3 sequence and orientation of VH3-33 mAbs influence peptide N-core conformation (A and B) X-ray crystal structures of seven 1210-like mAbs (A) and three MGG4-like mAbs (B) bound to various peptides, with C-core colored gray and N-core shown in green (A) or orange (B). HCDR1s, HCDR2s, KCDR1s, and KCDR3s are shown in gray, and HCDR3s are colored by mAb. The inset in (A) highlights the N-core type I β turn of the 1210-like binding conformation. The inset in (B) highlights the extended structure of the MGG4-like peptide N-core. (C) HCDR3 features that influence the N-core conformation are shown for representative 1210-like mAbs (N-core colored green) and MGG4-like mAbs (N-core colored orange) with Vk1-5 or non-Vk1-5 KCs, as indicated. KCs are shown in paler colors for each mAb. Fab-peptide complexes and notable residues are labeled. Fab-peptide salt bridges and HBs are shown as orange and yellow dashed lines, respectively. (D) X-ray crystal structure of Ky15.11-S^100I^K in complex with the KQPA peptide. Fab and peptide are colored as in (A), with mutated residue shown in red. The inset highlights the salt bridge (orange dashed line) between mutated H-Lys^100I^ and peptide Asp9. Electron density associated with the salt bridging residues is shown as blue mesh. (E) KQPA peptide bound by Ky15.11-S^100I^K is represented as sticks with residues labeled and corresponding electron density shown as blue mesh. (A and E) Blue dashed lines indicate an intrachain HB between main-chain atoms, and red dashed lines indicate an intrachain HB mediated by side-chain atoms. (D and E) Composite omit map electron density is contoured at 1.0 sigma.

**Figure 5:**
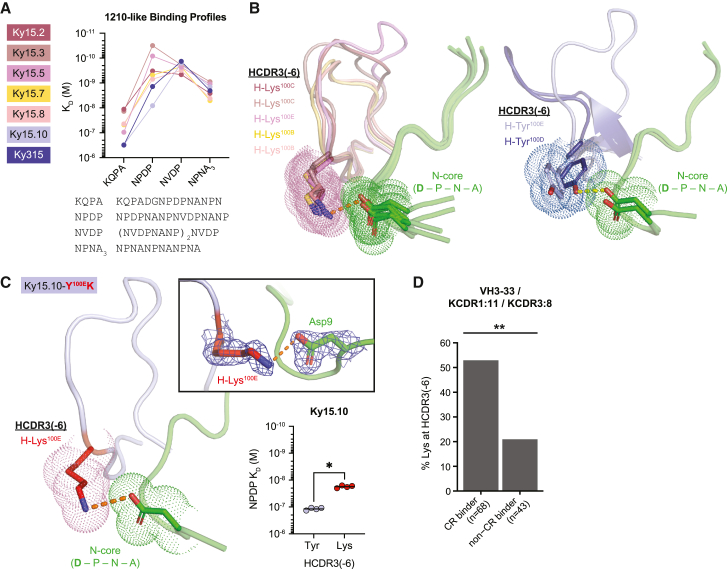
A specific Lys in the HCDR3 of 1210-like mAbs mediates cross-reactivity to junctional motifs (A) SPR binding profiles of 1210-like mAbs to the indicated peptides derived from the PfCSP junction and repeat regions. Binding profiles are colored by mAb according to the legend on the left, and peptide sequences are listed below. Data represent the mean of three independent measurements. (B) Non-covalent interactions facilitated by Lys and Tyr residues at position HCDR3(-6). Fab HCDR3s are colored by mAb based on the legend in (A), and peptides are shown in green. Interacting residues are depicted as stick representation with surrounding dots. Salt bridges are indicated by the orange dashed line, and the Ky315-DND HB is shown as a yellow dashed line. (C) X-ray crystal structure of Ky15.10-Y^100E^K in complex with the KQPA peptide. Fab and peptide are colored as in (B), with mutated residue shown in red. The top right inset highlights the salt bridge (orange dashed line) between mutated H-Lys^100E^ and peptide Asp9. The composite omit map electron density associated with the salt-bridging residues is shown as blue mesh and contoured at 1.0 sigma. Bottom right: Ky15.10 (HCDR3(-6) Tyr, light blue symbols) and Ky15.10-Y^100E^K (HCDR3(-6) Lys, red symbols) binding affinity to NPDP peptide as measured by SPR. Symbols represent independent measurements, and the black bar indicates geometric mean. Statistical significance was determined by two-tailed Mann-Whitney test (n = 4): ^∗^p < 0.05. (D) Frequency of Lys at position HCDR3(-6) among VH3-33/KCDR1:11/KCDR3:8 mAbs. n indicates the number of mAbs. Cross-reactive (CR) binders were defined as mAbs that bound at least three peptides by ELISA (Figure S1F). Statistical significance was determined using two-proportion Z test: ^∗∗^p < 0.01.

To gain insights into the molecular details of PfCSP recognition by these cross-reactive mAbs, we solved 20 X-ray crystal structures of the 12 Fab fragments in complex with various peptides derived from the PfCSP junction and central repeat regions (Figure 2; Table S3. X-ray crystallography data collection and refinement data for 1210-like mAbs and variants, related to Figures 2–5, Table S4. X-ray crystallography data collection and refinement data for MGG4-like mAbs and variants, related to Figures 2–4, Table S5. X-ray crystallography data collection and refinement data for Ky230 and Ky224, related to Figures 2 and 7). As in prior studies establishing the (N/D)PNANPN(A/V) sequence as the core epitope recognized by VH3-33 mAbs,^21^^,^^27^ we observed this core PfCSP epitope bound by the 12 mAbs characterized here. This motif can be further broken down into two N/D-P-N-A/V units that consistently form distinct secondary structural elements in the antibody-bound state.^15^^,^^17^^,^^27^^,^^28^ These structural units are here termed the N-terminal core (N-core) and C-terminal core (C-core) to reflect their sequential positions within the antibody-bound peptide (Figures 2 and 3A).

We found that the peptides bound by 11 of the 12 selected mAbs all adopted the same Asx turn (also denoted as an Asn pseudo 3_10_ turn) in the C-core^27^^,^^38^ and that this conformation was stabilized by common features that occurred in the heavy-chain complementarity-determining regions (HCDRs) despite varying degrees of somatic hypermutation (SHM; Table S2). For instance, mediated by intrachain hydrogen bonds (HBs), the HCDR1s and HCDR2s of all 11 mAbs exhibited identical secondary structural folds that supported key Fab-peptide interactions described previously^15^^,^^16^^,^^21^^,^^22^^,^^31^ (Figure 3B). In addition to these structural features, the HCDR2s of all 11 mAbs contained the following sequence composition, with germline (GL) residues largely maintained: Trp^52^(GL) - Tyr/Phe/His^52A^(GL Tyr^52A^) - Asp^53^(GL) - Gly^54^(GL) - Gly/Ser^55^(GL Ser^55^). In parallel, the HCDR1s and HCDR2s of these mAbs consisted of converging sites of SHM, most notably at positions 31 and 50, sites where SHM has been identified previously to improve VH3-33 mAb PfCSP repeat affinity.^22^ Strikingly, a shared feature also occurred within the highly variable HCDR3s of these 11 mAbs, which spanned 13–22 amino acids (aa) in length (Figure S3). Specifically, all 11 mAbs contained an HCDR3 residue that stacked against the Asx side chain, facilitating the C-core turn (Figures 3C and 3D). This feature appeared to play a defining role for the C-core conformation because Ky224 lacked an HCDR3 residue contributing such contacts to the peptide, resulting in an altered conformation with the Asp side chain pointing toward the N-core rather than the downstream Asn residue (Figure 3E). For 10 of the 11 mAbs that maintained the shared C-core conformation, these interactions were mediated by a Tyr residue (Figure 3C), whereas in the case of Ky230, the interacting HCDR3 residue was a Ser (Figure 3D). Because Ky230 exhibited the lowest level of liver burden reduction of the 12 mAbs (Figures 1F and 1H), this suggests that, in the context of this common C-core conformation, the presence of a Tyr residue stacking against the first Asx residue of the C-core contributes to effective parasite inhibition by VH3-33 mAbs. In summary, here we describe common HCDR features in 11 mAbs from clonally distinct B cell lineages that support a shared conformation in the C-core of the epitope, including specific interactions contributed by an HCDR3 Tyr that are associated with potent mAb function.

### N-core peptide conformation is defined by HCDR3 sequence and orientation

The 10 mAbs that exhibited high affinity for the major repeat and varying degrees of cross-reactivity (Figure 1C; Table S2) induced two distinct conformations in the antigen. The majority of mAbs (Ky15.2, Ky15.3, Ky15.5, Ky15.7, Ky15.8, Ky15.10, and Ky315) bound their respective peptides in an inverted S-shaped conformation identical to that elicited by mAb 1210 and several other VH3-33 human mAbs^15^^,^^21^^,^^22^^,^^23^^,^^27^^,^^39^ (Figure 4A; Table S3). This binding conformation consists of a type I β turn in the N-core of the peptide that is often strengthened by a side chain HB mediated by the first Asx residue (Figure 4A). Conversely, mAbs Ky15.1, Ky15.11, and Ky311 induced a fully extended N-core conformation without any secondary structural elements, similar to the human anti-PfCSP VH3-33 mAb MGG4^16^ (Figure 4B; Table S4).

Comparison of the 1210- and MGG4-like binding modes revealed that the N-core conformation was largely dictated by the HCDR3. All seven 1210-like mAbs contained a bulky Lys or Tyr residue six positions from the end of the HCDR3 (termed here position HCDR3(-6)) that directly occluded the MGG4-like conformation through steric hindrance (Figure 4C). The presence of an HCDR3(-6) Lys/Tyr appeared to correlate with *IGHJ* gene usage because all seven 1210-like mAbs were encoded by *IGHJ6^∗^02*, where Tyr is the GL-encoded HCDR3(-6) residue^40^ (Table S2). In contrast, the MGG4-like mAbs used *IGHJ4^∗^02* or *IGHJ5^∗^02* (Table S2), shorter gene segments that do not encode an HCDR3(-6) residue,^40^ and did not contain this steric occlusion, either due to having a small aa in this position (i.e., H-Ser^100I^ in Ky15.11) or because the overall orientation of the HCDR3 was angled away from the N-terminal end of the peptide (as is the case for Ky15.1 and Ky311; Figure 4C). In fact, when a Ser-to-Lys mutation was introduced at position HCDR3(-6) in Ky15.11 (Ky15.11-S^100I^K), the MGG4-like mAb was converted to a 1210-like binder, as shown by X-ray structure determination of the mutated Fab in complex with the KQPA peptide (Figures 4D and 4E; Table S4). In addition to the change in binding conformation, we also noted a register shift in the peptide as Ky15.11-S^100I^K bound the KQPA peptide with DPNA in the N-core like all other 1210-like mAbs crystallized with the KQPA peptide (Ky15.2, Ky15.8, and Ky15.10), whereas the wild-type Ky15.11 mAb positioned this motif in the C-core like the other MGG4-like mAbs (Figures 2 and 4E). Although the structural basis of this phenomenon remains unclear, our structure of Ky15.11 in complex with NDN reveals that MGG4-like binding is possible with the NPNV motif in the C-core rather than DPNA (Figure 2). Moreover, Vk1-5 mAbs as well as those using other KC genes were able to bind their peptides in either the 1210- or MGG4-like recognition modes, suggesting that different light chains can adapt to support either conformation. Thus, our structural studies identified two antigen conformations associated with high-affinity, cross-reactive binding and uncovered antibody properties that contribute to the underlying molecular basis of each binding mode.

### A specific HCDR3 Lys residue mediates cross-reactivity in the 1210-like binding mode

Despite the shared PfCSP recognition mode, the binding affinity profiles of the seven 1210-like mAbs were distinct, particularly for the KQPA (1.13 × 10^−8^ to 3.13 × 10^−7^ M) and NPDP peptides (3.14 × 10^−11^ to 8.14 × 10^−9^ M; Figure 5A). We observed that these differences in affinity were associated with the specific aa situated in the HCDR3(-6) position. In addition to its aforementioned role in shaping peptide conformation, this HCDR3 residue also formed important contacts with the first residue of the N-core. For five of the seven 1210-like mAbs (Ky15.2, Ky15.3, Ky15.5, Ky15.7, and Ky15.8), this position was occupied by a Lys residue that formed a salt bridge with the Asp at the beginning of the N-core when DPNA was bound in this site (Figure 5B). The remaining two 1210-like mAbs (Ky15.10 and Ky315) contained a Tyr at HCDR3(-6) that was only found to contribute HB interactions in Ky315 (Figure 5B). Ky15.10 and Ky315 had lower binding affinities to junctional peptides KQPA and NPDP compared with the five 1210-like mAbs with an HCDR3(-6) Lys residue (Figure 5A). This suggests that the Lys residue facilitates high-affinity binding to junctional epitopes where DPNA is present in the N-core, which aligns with the register by which the 1210-like mAbs bind KQPA and NPDP (Figure 2). Indeed, by substituting the *IGHJ*-encoded HCDR3(-6) Tyr residue of Ky15.10 for a Lys (Ky15.10-Y^100E^K), we confirmed the formation of a salt bridge without a register shift through X-ray crystallography of the mutated Fab in complex with KQPA and observed a significant enhancement in affinity for the NPDP peptide via SPR (Figure 5C; Table S3).

To determine whether HCDR3(-6) Lys was associated with cross-reactivity in the context of the 1210-like binding mode among a larger sample, we conducted a sequence analysis on the VH3-33 mAbs isolated in this study that shared common KC features with all seven 1210-like mAbs characterized here; namely, an 11-aa-long KCDR1 and 8-aa-long KCDR3. Of 148 VH3-33 mAbs, 111 contained these features, including mAbs paired with KCs encoded by the *IGKV1-5*, *IGKV1-12*, *IGKV3-11*, *IGKV3-15*, and *IGKV3D-15* gene segments. Within this subset, Lys at position HCDR3(-6) was significantly enriched among cross-reactive mAbs that bound three or more peptides in ELISA compared with non-cross-reactive mAbs (Figure 5D). Notably, all of the HCDR3(-6) Lys-containing cross-reactive mAbs identified here were encoded by the *IGHJ6* gene (Table S1). Thus, our data indicate that Lys at position HCDR3(-6) of 1210-like VH3-33 PfCSP mAbs is strongly associated with *IGHJ6* gene usage and plays a key role in mediating high-affinity cross-reactive binding.

### 1210-like binding is associated with high cross-reactivity and potent mAb efficacy

**Figure 6:**
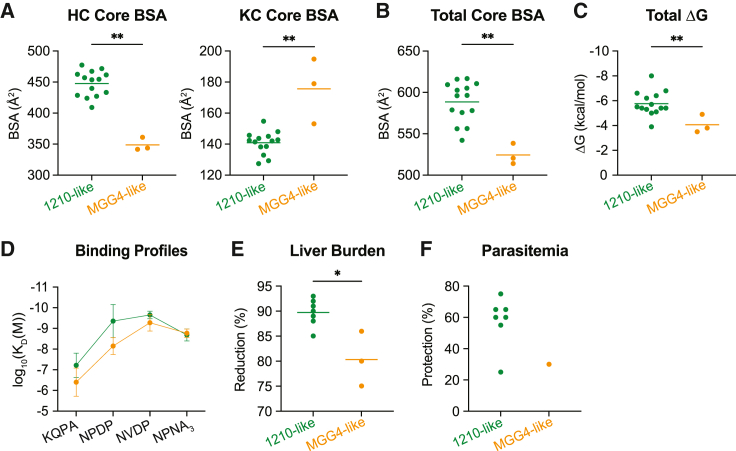
1210-like and MGG4-like binding conformations are associated with functional differences (A) Buried surface area (BSA) between the core epitope and heavy chain (HC; left) or kappa light chain (KC; right) of 1210- and MGG4-like mAbs. (B) Total BSA between Fabs and core epitope for 1210- and MGG4-like mAbs. (C) Theoretical change in free energy (ΔG) associated with binding of 1210- and MGG4-like mAbs to peptides based on crystallized Fab-peptide complexes. (D) SPR binding profiles of 1210- and MGG4-like mAbs to indicated peptides, represented as log K_D_ values. Error bars indicate standard deviation. (E and F) *In vivo* liver burden reduction (E) and parasitemia protection (F) conferred by 1210- and MGG4-like mAbs. (A–C and E) Horizontal lines represent arithmetic mean. n = 14 for 1210-like mAbs and n = 3 for MGG4-like mAbs (A–C); n = 7 for 1210-like mAbs and n = 3 for MGG4-like mAbs (E). Statistical significance was determined by two-tailed Mann-Whitney test: ^∗^p < 0.05, ^∗∗^p < 0.01.

We next sought to investigate whether the 1210-like and MGG4-like binding modes were associated with functional differences. To this end, we first examined the buried surface area (BSA) of each crystallized Fab-peptide complex for the 10 high-affinity mAbs, focusing only on the “core” epitope that consists of the full N-core and the first three residues of the C-core.^21^ These residues were resolved in all structures except one (Figure 2; Ky15.11 in complex with NDN, which was excluded from the analysis) and accounted for 73.7%–94.7% of total BSA in each structure. Although both binding modes were mainly mediated by the antibody HC (55.9%–73.4% of the total BSA), we observed that 1210-like mAbs had significantly greater BSA contributed by the HC compared with MGG4-like mAbs, whereas MGG4-like mAbs had more BSA contributed by the KC compared with 1210-like mAbs (Figure 6A). This aligns with our molecular description of the two binding modes because the bulky HCDR3(-6) Lys/Tyr residues that mediate 1210-like binding contributed a substantial amount of BSA, but in the absence of these residues, the peptide adopted the MGG4-like conformation, extending toward the KC. Overall, the 1210-like recognition mode resulted in significantly greater total BSA than the MGG4-like configuration among the crystallized Fab-peptide complexes examined here (Figure 6B). This difference was reflected in the theoretical free energy of binding (ΔG) as the 1210-like mAbs were typically predicted to induce a more favorable change in free energy upon peptide binding compared with those that bound in the MGG4-like disposition (Figure 6C). 1210-like binding also generally led to greater cross-reactivity of mAbs, with higher affinity to junctional peptides KQPA and NPDP (Figure 6D).

To determine whether these distinctions between the two PfCSP recognition modes were associated with differences in potency, we compared the *in vivo* efficacy of 1210-like mAbs with that of MGG4-like mAbs. Between seven 1210-like mAbs and three MGG4-like mAbs, we observed that the 1210-like mAbs exhibited significantly greater inhibition of parasite liver burden than the MGG4-like mAbs (Figure 6E). Similarly, among the limited number of mAbs evaluated for inhibitory efficacy against parasitemia, six 1210-like mAbs demonstrated 50% or greater inhibition, while the one MGG4-like mAb that was assessed conferred only 30% inhibition (Figure 6F). However, the 1210-like mAb Ky315 exhibited potency against parasitemia comparable with the MGG4-like mAb Ky15.1, indicating that 1210-like binding alone is not sufficient to confer high inhibitory capacity. In conclusion, our characterization of 10 high-affinity, cross-reactive *IGHV3-33*-encoded mAbs identified distinctions between the 1210-like and MGG4-like binding modes that were associated with differences in mAb cross-reactivity and *in vivo* functionality.

### A unique PfCSP binding conformation is associated with potent sporozoite inhibition

**Figure 7:**
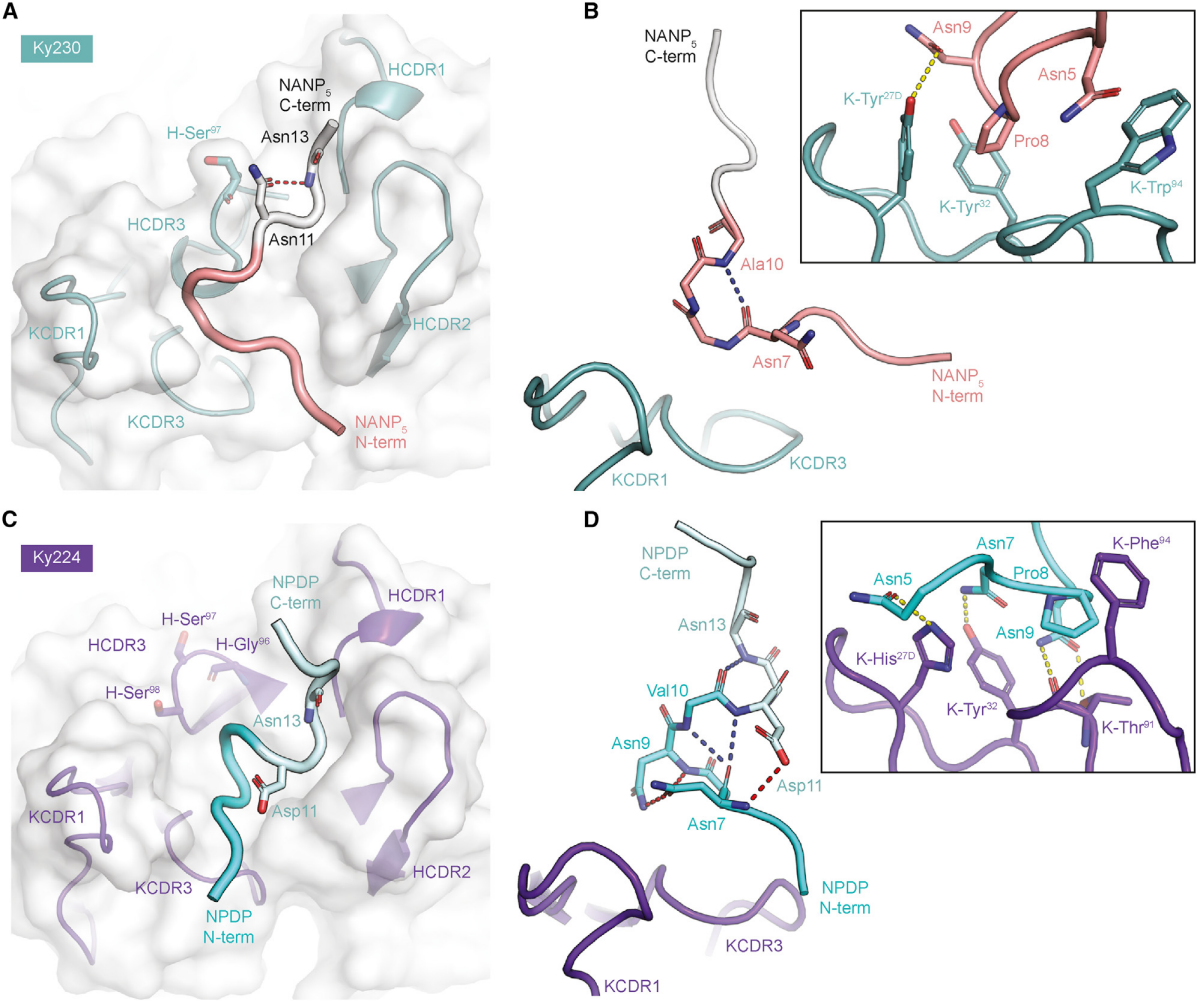
PfCSP peptides adopt unique conformations when bound to Ky230 and Ky224 mAbs (A) X-ray crystal structure of Ky230 Fab (white surface, teal cartoon) in complex with NANP_5_ (N-core colored salmon, C-core colored light gray). CDRs involved in binding, peptide termini, and notable residues are labeled. (B) Ky230-bound NANP_5_ N-core conformation with surrounding KCDR1 and KCDR3 loops. The inset highlights interactions contributed by aromatic KCDR1 and KCDR3 residues. (C) X-ray crystal structure of Ky224 Fab (white surface, purple cartoon) in complex with NPDP (N-core colored cyan, C-core colored pale cyan). CDRs involved in binding, peptide termini, and notable residues are labeled. (D) Ky224-bound NPDP N-core conformation with surrounding KCDR1 and KCDR3 loops. The inset highlights interchain HBs and CH/π interactions contributed by KCDR1 and KCDR3 residues. (A–D) Intrachain HBs between main-chain atoms are indicated by blue dashed lines, and those mediated by side-chain atoms are colored red. Fab-peptide HBs are shown as yellow dashed lines.

Interestingly, structural determination of Ky230 and Ky224, two mAbs with low, micromolar affinity for NPNA_3_ (Figure 1C; Table S2), revealed two previously unreported binding conformations for the PfCSP repeat motifs (Table S5). Encoded by the *IGKV2-30* and *IGKV2-24* genes, both mAbs contain 16-aa KCDR1s and 9-aa KCDR3s as well as the two shortest HCDR3s of the 12 VH3-33 mAbs (Table S2). Each of these mAbs bound their respective peptide in a distinct conformation, largely driven by differences in the HCDR3s. Ky230 induced the formation of an Asx turn in the peptide C-core, supported by stacking interactions contributed by H-Ser^97^ (Figure 7A). A type I β turn was also observed in the N-core of the Ky230-bound peptide between Asn7 and Ala10 (Figure 7B). In contrast to the 1210-like conformation, the Ky230-induced β turn was positioned toward the KCDR1 and stabilized by KCDR1 and KCDR3 aromatic residues (Figure 7B). Conversely, the HCDR3 of Ky224, shorter than that of Ky230 by three residues (Table S2), was oriented away from the binding groove, leaving H-Ser^97^ devoid of any peptide interactions (Figure 7C). Without these HCDR3 contacts, extra turns facilitated by several intrapeptide HBs occurred in the N-core of the epitope, altering the downstream C-core conformation (Figure 7D). KCDR1 and KCDR3 residues of Ky224 further stabilized the N-core conformation through HBs and aromatic stacking interactions (Figure 7D). Importantly, even though Ky230 and Ky224 exhibited similar peptide binding affinity profiles and comparable affinities for PfCSP (Figures 1C and 1D), passive immunization with Ky224 resulted in a 93% reduction in parasite liver load and 50% protection against parasitemia, comparable with the 1210-like mAbs, whereas Ky230 only reduced the liver burden by 62% (Figures 1F–1H). Thus, we elucidated two so far unreported binding conformations adopted by VH3-33 mAbs paired with non-Vk1-5 KCs with extended KCDRs, one of which mediated potent protection against parasite liver invasion despite demonstrating low cross-reactivity for the major NANP repeat.

## Discussion

Antibody correlates of protection are highly sought after to guide discovery and development efforts of new antibody-based interventions against Pf malaria. This goal is especially pertinent because mAbs against the PfCSP junction and central repeats have now been shown to be protective against Pf infection in pre-clinical and clinical models.^15^^,^^17^^,^^18^^,^^20^^,^^21^^,^^27^^,^^41^^,^^42^ Recent studies have reported a trend between the dissociation rate of PfCSP binding (*k*_*off*_) and mAb-mediated inhibition of parasite liver invasion.^27^^,^^39^ Others have shown that, although high affinity is required for sporozoite neutralization, mAb binding affinity is not well correlated with protection at high levels.^20^^,^^21^ Our study supports this view because we found that univariate *in vitro* measurements alone were not reliably predictive of *in vivo* efficacy among potent, high-affinity mAbs with greater than 75% liver burden reduction or 50% parasitemia inhibition. In fact, between 10 mAbs that exhibited comparable PfCSP binding affinity and *in vitro* sporozoite traversal inhibition, we discovered that differences in *in vivo* protective capacity were associated with distinct PfCSP recognition modes that mediated different levels of cross-reactivity. Specifically, we showed that the 1210-like binding mode confers stronger cross-reactivity and inhibitory efficacy than the MGG4-like configuration. We also noted that 1210-like mAbs bind peptides derived from the PfCSP junction (KQPA and NPDP) with a preferred register that positions the major NANP repeat in the center of the epitope between the N- and C-core motifs. These findings align with previous studies identifying antibody-induced epitope conformations consisting of regular secondary structural elements and high affinity to a core epitope centered around the major NANP repeat as features of protective PfCSP mAbs.^21^^,^^27^ Altogether, these data indicate that a high-resolution understanding of the molecular basis of cross-reactivity is particularly insightful.

Through extensive crystallography studies of 22 antibody-antigen complexes, we identified the aa at position HCDR3(-6) as a key residue amongst VH3-33 mAbs, influencing the PfCSP recognition mode and the binding affinity profile of the mAb. In particular, we demonstrate that the MGG4-like conformation induced by Ky15.11 was enabled by a small Ser in position HCDR3(-6). With a single point mutation of this residue to Lys, we were able to alter this mAb to adopt a 1210-like binding conformation. In this way, the size of the aa at position HCDR3(-6) may be indicative of the VH3-33 mAb recognition mode. In addition, building on a previous study where we established the electrostatic potential of the paratope as a key determinant of cross-reactivity through structural analysis of five 1210-like mAbs,^21^ we showed that having a Lys in the HCDR3(-6) position facilitates strong cross-reactivity among 1210-like mAbs. Beyond the current study, we also found this key HCDR3 Lys residue in the same structural position within previously characterized mAb 2541,^21^ a Vk1-5 mAb that indeed exhibited cross-reactive binding in the 1210-like recognition mode (Table S2). Because mAb 2541 is encoded by the *IGHJ3^∗^02* gene, which, similarly to *IGHJ4^∗^02* and *IGHJ5^∗^02*, is shorter than the *IGHJ6^∗^02* segment and does not encode an HCDR3(-6) residue,^40^ this indicates that this key Lys residue can arise through VDJ recombination and/or affinity maturation within non-*IGHJ6*-encoded 1210-like mAbs. However, as exhibited by Ky15.1 and Ky311, because of the position of this residue toward the end of the HCDR3, its impact is dependent on the fold and orientation of the HCDR3 loop. As antibody structure prediction algorithms continue to advance toward generating more accurate and high-throughput models,^43^ our description of molecular features underlying cross-reactive PfCSP recognition associated with potent mAb efficacy can be used to guide future antibody discovery and down-selection efforts and contribute to sequence-based evaluation of antibody responses elicited by next-generation immunogens.^35^

In contrast to the HC, the impact of the KC on binding conformation was less apparent from the current dataset. Although we show that the distinction between 1210-like and MGG4-like binding was not dependent on Vk gene usage, of the eight VH3-33/Vk1-5 mAbs reported here, six induced the 1210-like conformation, suggesting that mAbs with this gene combination may be predisposed to preferentially adopt this binding mode. This predisposition may in part be due to the Vk1-5 GL residue Trp^32^, which forms favorable stacking interactions with the HCDR3(-6) Lys/Tyr in the context of the 1210-like conformation (Figure 4C). However, because of the relatively small number of non-Vk1-5 mAbs in this study, additional work is needed to fully uncover the role of the light chain in cross-reactive PfCSP recognition.

By conducting this study using the Kymouse platform, we were able to examine human mAbs elicited by our novel immunogens and gain insights applicable to iterative vaccine design. For example, of the 15 different 1210-like VH3-33 mAbs that have been described in the literature so far (311;^15^ 1210;^22^ 2243, 2541, 4498, 3945, 3246;^21^ 356, 364, 395, 239;^27^ 334, 227, 337;^39^ and 850^23^), mAb 2541 was the only one to contain the key HCDR3 Lys described in this study. Although the relatively low frequency of this Lys residue among previously characterized human mAbs suggests that this may be a rare occurrence, this also highlights a potential functional advantage conferred by the junction-containing PfCSP repeat-based nanoparticle immunogens used in this study. In line with this, a recent investigation more broadly characterizing the antibody response elicited by the junction-NANP_5_ *H. pylori* Ferr nanocage revealed that immunization of mice from the Kymouse platform with this immunogen elicited significantly higher IgG titers against the PfCSP major repeat and junction compared to immunization with recombinant PfCSP, with evidence of affinity maturation and selection of SHMs known to facilitate high affinity.^35^ Further investigation will be needed to characterize the antibody response elicited by nanoparticle immunogens presenting cross-reactive epitopes in the context of the complete human Ig repertoire diversity. Together, our findings suggest that, to effectively leverage the highly prevalent *IGHV3-33*-encoded anti-PfCSP human antibody repertoire and induce a protective humoral response, rational immunogen design specifically aimed at eliciting high-affinity, cross-reactive 1210-like mAbs would be beneficial.

Recent efforts to enhance the protective efficacy of anti-PfCSP mAbs through phage display, inferred GL maturation, and *in silico* optimization have shown promise, showcasing the potential for antibody engineering to improve naturally acquired potent mAbs.^44^^,^^45^^,^^46^ We isolated and characterized Ky224, which adopted a so far unreported binding mode and demonstrated liver burden inhibition comparable with the best 1210-like mAbs studied here despite exhibiting relatively low affinity for PfCSP and poor cross-reactivity to the major NANP repeat. In contrast to the 1210-like mAbs that bound the NPDP peptide with the NANP motif positioned between the N- and C-core, Ky224 induced an NPDP peptide configuration centered on the NVDP motif (Figure 2), similar to neutralizing mAb L9.^18^^,^^39^^,^^42^ However, while mAb L9 accommodates the side chain of the central Val residue in a hydrophobic pocket formed by Vk1-5-encoded KCDR1, KCDR3, and HCDR3 residues,^39^ the Ky224-bound Val is surface exposed, contributing only ∼4 Å^2^ of BSA. Consequently, the structural basis for the NVDP-centered preference of Ky224 remains unclear, and additional studies are needed to develop a stronger understanding of this binding mode. Partly due to the low frequency of this KC gene,^47^^,^^48^ anti-PfCSP mAbs encoded by the *IGHV3-33/IGKV2-24* gene pairing are largely unexplored, with our structure of Ky224 in complex with the NPDP peptide being the first structural description of such a mAb. Overall, antibodies of this gene combination now represent a potential reservoir for novel insights into the protective human humoral response against PfCSP and a promising starting point for antibody engineering to deliver higher potency.

In conclusion, we provide a high-resolution structural description of molecular features for high-affinity, cross-reactive VH3-33 antibodies that are associated with potent Pf inhibition. We show that, within the mAb dataset characterized, the 1210-like binding mode is associated with stronger cross-reactivity and *in vivo* inhibitory capacity compared with the MGG4-like conformation and that this is largely influenced by a residue at the base of the HCDR3. These insights have implications in the context of efforts for the discovery, screening, and engineering of the most potent anti-PfCSP mAbs as well as the design of a next-generation anti-infective malaria immunogen aiming to elicit a protective cross-reactive antibody response against the PfCSP major repeats, and junctional and minor motifs. More broadly, the large structural dataset provided here will contribute to the growing collection of information that can be leveraged to guide *in silico* modeling efforts toward more accurate and precise predictions, particularly as it relates to features of the vast sequence and structural space of antibody HCDR3s.

### Limitations of study

While we provide a comprehensive structural description of the molecular basis of cross-reactivity and the association of binding conformation with *in vivo* mAb potency, many aspects of mAb-mediated parasite inhibition require further investigation. Anti-PfCSP antibodies have been shown to neutralize sporozoites through various mechanisms along the parasite’s journey through the skin, blood, and liver.^18^^,^^20^^,^^49^^,^^50^^,^^51^^,^^52^^,^^53^ Nonetheless, as antibody mode of action was not examined here, the impact of cross-reactivity and binding conformation on different mechanisms remains to be explored; likely through the use of sporozoite imaging and additional *in vivo* challenge models that more accurately reflect different aspects of sporozoite infection (e.g., inhibition of liver stage development assays using Pf sporozoites).^18^^,^^20^^,^^53^^,^^54^ Similarly, it remains unclear whether an antibody’s recognition mode influences its persistence in sera. Indeed, we noted that the titers of two passively transferred MGG4-like mAbs, Ky15.1 and Ky311, were markedly reduced in animal sera prior to *in vivo* PbPfCSP challenge. Additional work is thus needed to delineate whether there is any relationship between cross-reactivity, antibody recognition mode, and biodistribution more broadly. It is also worth noting that the binding of peptides captured by our X-ray crystal structures may not encompass the full scope of intricacies of mAb PfCSP recognition on the sporozoite surface. In fact, homotypic interactions and spiral ordering of PfCSP have been described in a monoclonal context by X-ray crystal structures with longer peptides containing two or more epitopes and cryoelectron microscopy studies with recombinant PfCSP.^22^^,^^23^^,^^27^^,^^29^^,^^30^^,^^55^ Whether these phenomena occur in a polyclonal setting remains to be seen. In the biological context of different Pf strains, the diversity of PfCSP repeat valency and motif organization as well as possible differences in PfCSP structural propensities may have varying effects on the functionality of cross-reactive mAbs with distinct binding modes. Because the work presented in this report was based on a single Pf strain (NF54), further examination will be required to elucidate the effects of cross-reactivity and antibody binding conformation on mAb functionality and breadth against PfCSP in its native state.

## STAR★Methods

### Key resources table

**Table:** 

REAGENT or RESOURCE	SOURCE	IDENTIFIER
**Antibodies**		

Goat anti-human IgG, Fcγ (HRP conjugated)	Jackson ImmunoResearch	Cat#109-035-098; RRID: AB_2337586

**Chemicals, peptides, and recombinant proteins**		

Ferr-junction-NANP_5_	This paper	N/A
Ferr-junction-NANP_18_	This paper	N/A
LS-junction-NANP_5_	This paper	N/A
LS-junction-NANP_18_	This paper	N/A
Sigma Adjuvant System (SAS)	Sigma Aldrich	Cat#S6322
KQPA	PSL GmbH, Heidelberg	N/A
NPDP	PSL GmbH, Heidelberg	N/A
DND	PSL GmbH, Heidelberg	N/A
NDN	PSL GmbH, Heidelberg	N/A
NANP_3_	PSL GmbH, Heidelberg	N/A
NPNA_3_	CPC Scientific	Product#924946
NANP_6_	CPC Scientific	Product#908957
KQPA	CPC Scientific	Product#926483
NPDP	CPC Scientific	Product#929317
NVDP	CPC Scientific	Product#929315
KQPA	GenScript	N/A
NPDP	GenScript	N/A
NANP_3_	GenScript	N/A
NDN	GenScript	N/A
DND	GenScript	N/A
NANP_5_	GenScript	N/A
mAbs listed in Table S1 isolated from the Kymouse™ platform expressed as IgG1	This paper	N/A
Ky15.1 Fab	This paper	N/A
Ky15.2 Fab	This paper	N/A
Ky15.3 Fab	This paper	N/A
Ky15.5 Fab	This paper	N/A
Ky15.7 Fab	This paper	N/A
Ky15.8 Fab	This paper	N/A
Ky15.10 Fab	This paper	N/A
Ky15.10- Y^100E^K Fab	This paper	N/A
Ky15.11 Fab	This paper	N/A
Ky15.11-S^100I^K Fab	This paper	N/A
Ky224 Fab	This paper	N/A
Ky230 Fab	This paper	N/A
Ky311 Fab	This paper	N/A
Ky315 Fab	This paper	N/A
NF54 PfCSP	This paper	N/A
GIBCO Freestyle 293 Expression Medium	Thermo Fisher Scientific	Cat#12338026
G418 (Geneticin)	Cedarlane	Cat#ANT-GN-1
FectoPRO DNA Transfection Reagent	VWR	Cat#10118-444
PEI MAX DNA Transfection Reagent	Polysciences	Cat#24765-100

**Critical commercial assays**		

Series S Sensor Chip CM5	GE Healthcare	Cat#BR100530
Human Antibody Capture Kit	GE Healthcare	Cat#BR100839
CMD200M Sensor Chip	Carterra	Cat#4280
Ni-NTA Biosensors	FortéBio	Cat#18-5102

**Deposited data**		

Crystal structure of Ky15.2 Fab in complex with circumsporozoite protein KQPA peptide	This paper	PDB: 8F9E
Crystal structure of Ky15.2 Fab in complex with circumsporozoite protein NPDP peptide	This paper	PDB: 8F9T
Crystal structure of Ky15.2 Fab in complex with circumsporozoite protein DND peptide	This paper	PDB: 8F95
Crystal structure of Ky15.2 Fab in complex with circumsporozoite protein NDN peptide	This paper	PDB: 8F9S
Crystal structure of Ky15.2 Fab in complex with circumsporozoite protein NANP_3_ peptide	This paper	PDB: 8F9F
Crystal structure of Ky15.3 Fab in complex with circumsporozoite protein NPDP peptide	This paper	PDB: 8FDD
Crystal structure of Ky15.5 Fab in complex with circumsporozoite protein NPDP peptide	This paper	PDB: 8FA9
Crystal structure of Ky15.7 Fab in complex with circumsporozoite protein NPDP peptide	This paper	PDB: 8F9U
Crystal structure of Ky15.8 Fab in complex with circumsporozoite protein KQPA peptide	This paper	PDB: 8F9V
Crystal structure of Ky15.8 Fab in complex with circumsporozoite protein NPDP peptide	This paper	PDB: 8F9W
Crystal structure of Ky15.10 Fab in complex with circumsporozoite protein KQPA peptide	This paper	PDB: 8FB6
Crystal structure of Ky15.10 Fab in complex with circumsporozoite protein NPDP peptide	This paper	PDB: 8FB7
Crystal structure of Ky15.10 Fab in complex with circumsporozoite protein DND peptide	This paper	PDB: 8FA6
Crystal structure of Ky15.10-Y^100E^K Fab in complex with circumsporozoite protein KQPA peptide	This paper	PDB: 8FB8
Crystal structure of Ky315 Fab in complex with circumsporozoite protein DND peptide	This paper	PDB: 8FBA
Crystal structure of Ky15.1 Fab in complex with circumsporozoite protein KQPA peptide	This paper	PDB: 8FAN
Crystal structure of Ky15.11 Fab in complex with circumsporozoite protein KQPA peptide	This paper	PDB: 8FA7
Crystal structure of Ky15.11 Fab in complex with circumsporozoite protein NDN peptide	This paper	PDB: 8FA8
Crystal structure of Ky15.11-S^100I^K Fab in complex with circumsporozoite protein KQPA peptide	This paper	PDB: 8FB5
Crystal structure of Ky311 Fab in complex with circumsporozoite protein KQPA peptide	This paper	PDB: 8FDC
Crystal structure of Ky230 Fab in complex with circumsporozoite protein NANP_5_ peptide	This paper	PDB: 8FAS
Crystal structure of Ky224 Fab in complex with circumsporozoite protein NPDP peptide	This paper	PDB: 8FAT

**Experimental models: Cell lines**		

Expi293F™ Cells	Gibco	Cat #A14635
CHO-3E7 Cells	NRC Canada	File 11992
Freestyle™ 293-F cells	Thermo Fisher Scientific	Cat#12338026
Human Hepatocyte HC-04 cells	BEI Resources	MRA-975

**Experimental models: Organisms/strains**		

Mice from the Kymouse™ platform	KymAb^34^	N/A
*P. falciparum* NF54 salivary gland sporozoites	Delemarre-van de Waal and de Waal^56^	N/A
C57BL/6 Mice	Charles River Laboratories	Cat#027
*P. berghei* sporozoites expressing PfCSP, GFP and luciferase	Flores-Garcia et al.^57^	N/A

**Recombinant DNA**		

pTT5 plasmids containing heavy and light chain variable genes of mAbs listed in Table S1 isolated from the Kymouse™ platform expressed as IgG1	NRC Canada	File 11266
pcDNA3.4-PfCSP-His	This paper	N/A
pcDNA3.4-Ky15.1-Fab-HC	This paper	N/A
pcDNA3.4-Ky15.1-Fab-KC	This paper	N/A
pcDNA3.4-Ky15.2-Fab-HC	This paper	N/A
pcDNA3.4-Ky15.2-Fab-KC	This paper	N/A
pcDNA3.4-Ky15.3-Fab-HC	This paper	N/A
pcDNA3.4-Ky15.3-Fab-KC	This paper	N/A
pcDNA3.4-Ky15.5-Fab-HC	This paper	N/A
pcDNA3.4-Ky15.5-Fab-KC	This paper	N/A
pcDNA3.4-Ky15.7-Fab-HC	This paper	N/A
pcDNA3.4-Ky15.7-Fab-KC	This paper	N/A
pcDNA3.4-Ky15.8-Fab-HC	This paper	N/A
pcDNA3.4-Ky15.8-Fab-KC	This paper	N/A
pcDNA3.4-Ky15.10-Fab-HC	This paper	N/A
pcDNA3.4-Ky15.10-Fab-KC	This paper	N/A
pcDNA3.4-Ky15.10-Y^100E^K -Fab-HC	This paper	N/A
pcDNA3.4-Ky15.11-Fab-HC	This paper	N/A
pcDNA3.4-Ky15.11-Fab-KC	This paper	N/A
pcDNA3.4-Ky15.11-S^100I^K-Fab-HC	This paper	N/A
pcDNA3.4-Ky315-Fab-HC	This paper	N/A
pcDNA3.4-Ky315-Fab-KC	This paper	N/A
pcDNA3.4-Ky311-Fab-HC	This paper	N/A
pcDNA3.4-Ky311-Fab-KC	This paper	N/A
pcDNA3.4-Ky230-Fab-HC	This paper	N/A
pcDNA3.4-Ky230-Fab-KC	This paper	N/A
pcDNA3.4-Ky224-Fab-HC	This paper	N/A
pcDNA3.4-Ky224-Fab-KC	This paper	N/A
pHL-*sec*-Ferr-junction-NANP_5_	This paper	N/A
pHL-*sec*-Ferr-junction-NANP_18_	This paper	N/A
pHL-*sec*-LS-junction-NANP_5_	This paper	N/A
pHL-*sec*-LS-junction-NANP_18_	This paper	N/A

**Software and algorithms**		

GraphPad Prism	GraphPad Software, LLC	https://www.graphpad.com/scientific-software/prism/
Biacore T200 Software	Cytiva	N/A
Kinetics Software	Carterra	https://carterra-bio.com/resources/kinetics-software/
TitrationAnalysis Tool	Li et al.^58^	https://github.com/DukeCHSI/TitrationAnalysis
Octet Data Analysis Software	FortéBio	http://www.fortebio.com/
XDS	Kabsch^59^	http://xds.mpimf-heidelberg.mpg.de/
Phaser	McCoy et al.^60^	https://www.phenix-online.org/
Phenix	Adams et al.^61^	https://www.phenix-online.org/
Coot	Emsley et al.^62^	https://www2.mrc-lmb.cam.ac.uk/personal/pemsley/coot/
PyMOL	Schrödinger, LLC	The PyMOL Molecular Graphics System, v2.3.4.
SBGrid	Morin et al.^63^	https://sbgrid.org/software/titles/sbgrid-installer
hbplus	McDonald and Thornton^64^	https://www.ebi.ac.uk/thornton-srv/software/HBPLUS/
PDBePISA	Krissinel and Henrick^65^	https://www.ebi.ac.uk/pdbe/pisa/
DSSP	Touw et al.^66^; Kabsch and Sander^67^	https://swift.cmbi.umcn.nl/gv/dssp/
Clustal Omega	Sievers et al.^68^	https://www.ebi.ac.uk/Tools/msa/clustalo/
R (version 4.2.3)	The R Foundation	https://www.r-project.org/foundation/

**Other**		

The International Immunogenetics Information System (IMGT)	Lefranc^40^	https://imgt.org/

### Resource availability

#### Lead contact

Further information and requests for resources and reagents should be directed to and will be fulfilled by the lead contact, Jean-Philippe Julien (jean-philippe.julien@sickkids.ca).

#### Materials availability

All unique and stable reagents generated in this study are available via the lead contact upon a reasonable request.

### Experimental model and study participant details

#### Mammalian cell lines and culture conditions

Expi293F cells from the Expi293 Expression System (Thermo Fisher Scientific, A14635) were cultured in suspension in GIBCO Expi293 Expression Medium (Thermo Fisher Scientific) at 37°C with 5% CO_2_ and standard humidified conditions.

CHO-3E7 cells from NRC Canada (NRC file 11992) were cultured in suspension in GIBCO FreeStyle F17 Expression Medium (Thermo Fisher Scientific, A1383501) supplemented with glucose (Thermo Fisher Scientific) and Kolliphor (BASF Pharma) at 37°C in an ISF1-X shaking incubator (Kuhner) with 5% humidity, 5% CO_2_.

FreeStyle 293-F cells (Thermo Fisher Scientific, 12338026) were cultured in suspension in GIBCO FreeStyle 293 Expression Medium (Thermo Fisher Scientific) at 37°C in a Multitron Pro Shaker (Infors HT) with 70% humidity, 8% CO_2_ and rotating at 130 rpm.

HC-04 cells (MRA-975) were cultured in DMEM/F-12 (Gibco 31330)/1% PenStrep (Gibco 15140-122)/10% hiFBS (Gibco 10270-106) in a humidified incubator at 37°C and 5% CO_2_. They were sub-cultured twice weekly.

#### Mice

Male and female transgenic mice from the Kymouse platform between the ages of 7–12 weeks at the time of immunization were housed and all procedures carried out under United Kingdom Home Office License 70/8718 with the approval of the Wellcome Trust Sanger Institute Animal Welfare and Ethical Review Body.

Female 6-7-week-old C57BL/6 mice were purchased from Charles River. One week after arrival to the animal facilities they were used for experiments. All animal procedures were approved by the Johns Hopkins ACUC with protocol number MO18H419. Since these experiments were done only with female mice, this might represent a limitation to the research generalizability.

#### Sporozoites

*Pf* NF54 asexual and sexual blood stage parasites were cultured in a semi-automated culture system as described by Ponnudurai.^69^^,^^70^ Sporozoites were produced by feeding *Anopheles stephensi* mosquitos (Sind-Kasur Nijmegen strain) using standard membrane feeding of cultured gametocytes, as was described by Feldmann.^71^ Salivary glands were dissected, collected in Leibovitz L15 medium supplemented with 1% PenStrep and homogenized in a homemade glass grinder. Sporozoites were counted in a Bürker-Türk counting chamber using phase-contrast microscopy.

*P. berghei* transgenic parasites expressing PfCSP, GFP and luciferase were generated as previously described.^57^ Briefly, *P. berghei*, strain ANKA 676m1c11, which expresses GFP and luciferase were obtained from MR4 (ATCC). Parasites were transfected to replace the CSP gene with a modified gene consisting of the CSP of Pf. To obtain sporozoites, *Anopheles stephensi* mosquitos were fed on mice infected with the parasite. 19–22 days later salivary glands from the infected mosquitos were obtained and sporozoites were isolated to infect mice.

### Method details

#### Mouse immunization

A mix of male and female transgenic mice from the Kymouse platform^34^ were immunized with Ferr-junction-NANP_5_, Ferr-junction-NANP_18_, LS-junction-NANP_5_ or LS-junction-NANP_18_ nanoparticles in the presence of Sigma Adjuvant System (SAS, Sigma Aldrich). Mice from the Kymouse platform contain chimeric immunoglobulin loci, with humanized variable domains (V_H_, V_K_, and V_L_) and a humanized lambda constant domain (C_L_), but murine heavy (C_H_) and kappa (C_K_) constant domains. Kymab and Kymouse are trademarks of Sanofi Group. Mice were injected subcutaneously at base of tail at weeks 0, 4 and 10. Mice were sacrificed 7 days after the final boost under UK Home Office Schedule 1 (rising concentration of CO_2_). Spleens, lymph nodes, and bone marrows from each mouse were collected, and single-cell suspensions cryopreserved in 10% DMSO/FBS were stored in liquid nitrogen until further analysis. Whole blood was collected 1 week after each dose (weeks 1, 5 and week 11 terminal bleed). Serum was separated from hematocrit via centrifugation at 2000*g* for 10 min, stored at −20°C and used to monitor titers by ELISA.

#### Antibody isolation

Cryopreserved single-cell suspensions were sorted by fluorescence-activated cell sorting (FACS) into individual wells of a 96-well plate in order to recover CD19^+^ B220^+^ GL7^+^ CD95^+^ germinal center B cells from spleen and lymph nodes as well as CD138^+^ TACI^+^ plasma cells from spleen, lymph node and bone marrow samples (BD FACS Aria Fusion flow cytometer, Beckton Dickinson). Paired Ig genes were amplified by RT-PCR and Illumina libraries were generated before sequencing on an Illumina MiSeq. Reads corresponding to the same plate/well location were combined into consensus sequences. Germline assignment and sequence annotation of the consensus sequences was performed as previously described.^34^ Heavy and light chain variable genes of selected antibodies were synthesized by Twist Biosciences (San Francisco, USA). Antibodies were recombinantly expressed as fully human IgG1 in HEK293 cells (Expi293F Cells, Gibco, Cat. No. A14635). Antibody supernatant was collected on day 8 after transfection and screened for binding by ELISA. Antibodies that bound PfCSP were re-expressed in suspension CHO-3E7 cells (NRC Canada) at a larger scale and purified using gravity flow columns (Econo-Pac Chromatography Columns, Bio-Rad, 732–1010) containing 1 mL MabSelect SuRe LX resin (GE Healthcare, 17-5474-03, P3303174) in order to generate enough material for further *in vitro* and *in vivo* assays. Antibody sequences were analyzed as previously described.^32^

#### CSP peptides

Synthetic peptides were custom made by Peptide Speciality Laboratory (PSL), Heidelberg (KQPA (KQPADGNPDPNANPN), NPDP (NPDPNANPNVDPNANP), DND (NVDPNANPNVDP), NDN (NANPNVDPNANP) and NANP_3_ (NANPNANPNANP)), CPC Scientific (NPNA_3_ (NPNANPNANPNA), NANP_6_ (NANPNANPNANPNANPNANPNANP), KQPA, NPDP and NVDP (NVDPNANPNVDPNANPNVDP)) or GenScript (KQPA, NPDP, NANP_3_, NDN, DND, and NANP_5_ (NANPNANPNANPNANPNANP)). With the exception of NANP_6_, all other peptides were acetylated at N-termini and amidated at C-termini. NANP_6_ contained an N-terminal biotin-aminohexanoic acid tag and an unmodified C terminus.

#### Enzyme-linked immunosorbent assay (ELISA)

Antigen ELISAs were performed as previously described.^21^^,^^25^ In brief, high-binding 384-well polystyrene plates (Corning) were coated overnight at 4°C with 2 μg/mL of the indicated peptides synthesized at PSL, Heidelberg. Plates were washed three times with 0.05% Tween 20 in PBS, blocked with 50 μL of 1% BSA in PBS for 1 h at room temperature (RT), and washed again. For analysis of mAbs, 15 μL/well of serially diluted mAbs (starting concentration 1.0 μg/mL, 1 in 4 dilution for 4 steps) were incubated for 2 h at RT. Wells were washed six times and incubated with goat anti-human IgG-HRP at a 1:1000 dilution (Jackson ImmunoResearch) in PBS with 1% BSA for 1 h. Wells were washed again and one-step ABTS substrate (RT, 20 μL/well; Roche) and 1× KPL ABTS peroxidase stop solution (RT, 20 μL/well; SeraCare Life Sciences) were used for detection. Area under the ELISA curve (AUC) was calculated using GraphPad Prism 7.04 (GraphPad).

#### Surface plasmon resonance (SPR)

Affinity of 56 VH3-33 mAbs to DND and NANP_3_ peptides were measured using SPR in Biacore Cytiva T200, as described before.^21^ In brief, series S sensor chip CM5 (GE Healthcare) was docked in the instrument and 10 mM HEPES (pH 7.4) containing 200 mM NaCl, 0.02% Tween 20 and 0.05% BSA was used as running buffer. Anti-human IgG from human antibody capture kit (GE Healthcare) was immobilized in both flow cells through amine coupling following manufacturer’s instructions. Equal concentrations of anti-PfCSP antibodies and non-PfCSP-reactive antibody (mGO53^72^) were captured in the sample and reference flow cells, respectively. Upon stabilizing flow cells with running buffer for 20 min, indicated peptides synthesized at PSL Heidelberg were injected at 0, 0.015, 0.09, 0.55, 3.3 and 20 μM concentration at a flow rate of 30 μL/min. Association and dissociation of the peptides were measured at 25°C for 60 s and 180 s, respectively. 3M MgCl_2_ was used for the regeneration of both flow cells. 1:1 binding model or steady-state kinetic analysis in the Biacore T200 software V2.0 was used for fitting the data and obtaining kinetic rates and constant.

For in-depth affinity characterization of the 12 selected mAbs, binding kinetics measurements were performed using the Carterra LSA high-throughput SPR platform and CMD200M sensor chips (Carterra) at 25°C as described before.^23^ In each assay, a single analyte peptide antigen (KQPA, NPDP, NVDP, NPNA_3_ or NANP_6_) was titrated against the immobilized mAbs. Using 10 mM MES buffer at pH 5.5 with 0.01% Tween 20 as running buffer, the chip was first activated by N-Hydroxysuccinimide (NHS) and 1-Ethyl-3-(3-dimethylaminopropyl) carbodiimide hydrochloride (EDC). Then, PfCSP-specific mAbs were directly immobilized (in 10 mM sodium acetate at pH 4.5) at 10 μg/mL or 5 μg/mL concentrations for 600 s, followed by quenching of unreactive esters using 1 M ethanolamine-HCl at pH 8.5. Then, 45 cycles of 1X HBSTE buffer (10 mM HEPES pH 7.4, 150 mM NaCl, 3 mM EDTA and 0.01% Tween 20) injections with 1X HBSTE also as running buffer were used to wash off non-specifically bound IgG on the chip surface. Each PfCSP-specific mAb at a given diluted concentration was immobilized onto 4 separate spots of the same chip, enabling replicate measurements.

A 2-fold dilution series of the peptide antigens was prepared in 1x HBSTE buffer, then injected onto the chip surface from the lowest to the highest concentration without regeneration following 8 injections of running buffer for signal stabilization. The top concentration for all PfCSP peptide antigens was 8 μg/mL (2.92 μM for NANP_6_, 6.41 μM for NPNA_3_, 3.76 μM for NVDP, 4.70 μM for NPDP, 5.03 μM for KQPA). For each concentration, the data collection involved 120 s of baseline step and 900 s of dissociation steps. The association step duration was 240 s for NANP_6_ and 300 s for all other PfCSP peptide antigens. The running buffer was 1X HBSTE for all titrations.

The kinetics titration data collected were first pre-processed in the Kinetics (Carterra) software, including reference subtraction, buffer subtraction and data smoothing. The data were then exported and analyzed using the TitrationAnalysis tool developed in-house.^58^ Each titration time course was fitted to a 1:1 Langmuir model to derive *k*_*on*_, *k*_*off*_ and *K*_*D*_ values. For antigens with multiple repeats of epitopes, the *K*_*D*_ values determined includes avidity effect. For each antibody–antigen pair, the mean kinetics estimates were calculated using the best triplicate measurements satisfying the pre-set data acceptance criteria: 1) standard error of the estimated *k*_*on*_, *k*_*off*_ and *K*_*D*_ in each replicate ≤20% and 2) fold change for *k*_*on*_, *k*_*off*_ and *K*_*D*_ values within the triplicate ≤3.

#### Recombinant PfCSP production and purification

A pcDNA3.4 expression vector was generated with a C-terminally His-tagged construct of full-length PfCSP isolated from strain NF54 (UniProt accession no. P19597, residues 20–375), and an internal ribosome entry site (IRES) followed by green fluorescent protein (GFP). The resulting plasmid was transfected into FreeStyle HEK293F cells and GFP fluorescence was used to select for single cells via FACS. The selected single cell was expanded and cultured in GIBCO Freestyle 293 Expression Medium and maintained with 200 μg/mL of geneticin (G418 solution; Invivogen). Protein expression was enabled by subculturing the stable cells without antibiotic to a density of 8 x 10^5^ cells/mL and harvested after 5–7 days. PfCSP was purified by HisTrap FF affinity chromatography (Cytiva) and size exclusion chromatography (Superdex 200 Increase 10/300 GL, Cytiva).

#### Biolayer interferometry (BLI)

BLI (Octet RED96, FortéBio) experiments were conducted to determine the binding kinetics of anti-PfCSP mAbs to recombinant full-length PfCSP. PfCSP was diluted to 10 μg/mL in kinetics buffer (PBS, pH 7.4, 0.01% [w/v] BSA, 0.002% [v/v] Tween 20) and immobilized onto Ni-NTA (NTA) biosensors (FortéBio). After a steady baseline was established, biosensors were dipped into wells containing 2-fold dilutions of each mAb in kinetics buffer. Tips were then immersed back into kinetics buffer for measurement of the dissociation rate. Kinetics data were analyzed using the FortéBio’s Octet Data Analysis software 9.0.0.6, and curves were fitted to a 1:1 binding model.

#### Traversal assay

Human hepatoma (HC-04) cells were seeded in microtiter plates and grown to near confluence. Fresh *P. falciparum* salivary gland sporozoites^56^ were isolated from *Anopheles stephensi* mosquitoes and pre-incubated with diluted IgG for 30 min before adding rhodamine dextran. Following incubation for 1 h at 37°C, cell nuclei were stained with DAPI. Fluorescence levels of traversed cells were quantitated using a high content automated imager.

#### Liver burden reduction assay

Parasite challenge to measure liver burden was assessed as previously described.^57^ Briefly, 6-8-week-old C57BL/6 female mice were injected intravenously with 100 μg of the respective mAb. Mice were challenged 16 h later with 2,000 PfCSP-expressing transgenic Pb sporozoites delivered i.v. in HBSS-2% FBS. Mice were injected intraperitoneally with 100 μL of D-luciferin at 30 mg/mL 42 h after sporozoite challenge, anesthetized with isoflurane and imaged in the IVIS Spectrum Imaging System to measure the bioluminescence expressed by the transgenic parasite.

#### Bite-parasitemia challenge

Assessment of sterile immunity conferred by mAbs was performed as previously described in detail.^57^ Briefly, 6-8-week-old female C57BL/6 mice received an injection of the respective mAb into the tail vein and 16 h later were exposed to the bites of five parasite-infected mosquitoes, selected from a population of mosquitoes that was 80% infected. Mice were anesthetized with 2% Avertin and placed on top of cages containing the infected mosquitoes for 10 min. Four days after challenge, Giemsa-stained blood smears were examined daily by light microscopy to assess the presence of blood-stage parasites from days 4–10 post-challenge. Protection from parasitemia was determined by the proportion of uninfected animals remaining after the observation period post-challenge. Results are shown in Kaplan-Meier curves.

#### Fab production

Fabs of mAbs Ky15.1, Ky15.2, Ky15.3, Ky15.5, Ky15.7, Ky15.8, Ky15.10, Ky15.10-Y^100E^K, Ky15.11, Ky15.11-S^100I^K, Ky224, Ky230, Ky311 and Ky315 were generated by cloning the *IGH* and *IGK* variable region gene segments into custom pcDNA3.4 expression vectors immediately downstream of a human Igκ signal peptide and upstream of human *CH1 and IGK* constant regions, respectively. pcDNA3.4-Fab HC and KC plasmids were co-transfected into HEK293F cells (Thermo Fisher Scientific) for transient expression using FectoPRO (Polyplus) or PEI MAX (Polysciences) DNA transfection reagents. Cells were cultured in GIBCO FreeStyle 293 Expression Medium for 5–7 days and purified via KappaSelect affinity chromatography (Cytiva) and cation-exchange chromatography (MonoS, Cytiva).

#### Crystallization and structure determination

Purified Fabs of mAb Ky15.2, Ky15.3 and Ky15.7 were concentrated and diluted to 10 mg/mL with various peptides in a 1:3 molar ratio in 20 mM sodium acetate pH 5.6 buffer. Ky15.2-NPDP co-crystals grew in 0.1 M CHES pH 9.5, 20% (w/v) PEG8000 and were cryoprotected in 15% (w/v) ethylene glycol after 11 days of crystal growth. Ky15.2-KQPA, Ky15.2-DND, Ky15.2-NDN and Ky15.2-NANP_3_ co-crystals grew after micro-seeding from thin, layered plate-like crystals that grew in 8.5% (w/v) isopropanol, 15% (w/v) glycerol, 17% (w/v) PEG4000, 0.085 M sodium HEPES pH 7.5. Ky15.2-KQPA co-crystals grew in 0.1 M HEPES pH 7.5, 25% (w/v) PEG3350, 0.2 M sodium chloride and were cryoprotected in 15% (w/v) ethylene glycol after 5 days of crystal growth. Ky15.2-DND co-crystals grew in 25% (w/v) PEG3350, 0.2 M NaCl, 0.1 M Tris pH 8.5 and were cryoprotected in 15% (w/v) ethylene glycol after 5 days of crystal growth. Ky15.2-NDN co-crystals grew in 0.2 M ammonium fluoride, 20% (w/v) PEG3350 and were cryoprotected in 15% (w/v) ethylene glycol after 5 days of crystal growth. Ky15.2-NANP_3_ co-crystals grew in 0.2 M magnesium acetate, 20% (w/v) PEG3350 and were cryoprotected in 15% (w/v) ethylene glycol after 5 days of crystal growth. Ky15.3-NPDP co-crystals grew in 0.1 M CHES pH 9.5, 30% (w/v) PEG8000 and were cryoprotected in 15% (w/v) ethylene glycol after 14 days of crystal growth. Ky15.7-NPDP co-crystals grew in 0.2 M di-ammonium hydrogen phosphate, 20% (w/v) PEG3350 and were cryoprotected in 15% (w/v) ethylene glycol after ∼60 days of crystal growth.

Purified Ky15.5 and Ky15.8 Fabs were concentrated and diluted to 11 mg/mL with various peptides in a 1:3 molar ratio in 20 mM sodium acetate pH 5.6 buffer. Ky15.5-NPDP co-crystals grew in 20% (w/v) PEG3350, 0.2 M tri-lithium citrate and were cryoprotected in 10% (w/v) ethylene glycol after ∼90 days of crystal growth. Ky15.8-KQPA co-crystals grew in 20% (w/v) isopropanol, 20% (w/v) PEG4000, 0.1 M tri-sodium citrate pH 5.6 and were cryoprotected in 10% (w/v) ethylene glycol after 5 days of crystal growth. Ky15.8-NPDP co-crystals grew in 0.2 M ammonium sulfate, 0.1 M HEPES pH 7.5, 25% (w/v) PEG3350 and were cryoprotected in 15% (w/v) ethylene glycol after 5 days of crystal growth.

Purified Ky15.10 Fab was concentrated and diluted to 6.7 mg/mL with KQPA and NPDP peptides in a 1:3 molar ratio in 20 mM sodium acetate pH 5.6 buffer and diluted to 12.3 mg/mL with DND peptide similarly. Ky15.10-KQPA co-crystals grew in 0.1 M sodium acetate pH 4.5, 40% (w/v) 1,2-propanediol and were cryoprotected in 15% (w/v) ethylene glycol after ∼50 days of crystal growth. Ky15.10-NPDP co-crystals grew in 0.2 M calcium chloride, 20% (w/v) PEG3350 and were cryoprotected in 10% (w/v) ethylene glycol after ∼50 days of crystal growth. Ky15.10-DND co-crystals grew in 0.2 M ammonium acetate, 0.1 M HEPES pH 7.5, 25% (w/v) PEG3350 and were cryoprotected in 10% (w/v) ethylene glycol after ∼90 days of crystal growth. Purified Ky15.10-Y^100E^K Fab was concentrated and diluted to 10 mg/mL with KQPA peptide in a 1:3 molar ratio in 20 mM sodium acetate pH 5.6 buffer. Ky15.10-Y^100E^K-KQPA co-crystals grew in 0.1 M HEPES pH 7.5, 20% (w/v) PEG8000 and were cryoprotected in 20% (w/v) glycerol after 24 days of crystal growth.

Purified Ky15.11 and Ky15.11-S^100I^K Fabs were concentrated and diluted to 10 mg/mL with KQPA peptide in a 1:3 molar ratio in 20 mM sodium acetate pH 5.6 buffer. Ky15.11-KQPA co-crystals grew in 15% (w/v) glycerol, 25.5% (w/v) PEG4000, 0.085 M tri-sodium citrate and were looped for X-ray diffraction experiments without additional cryoprotectant after 5 days of crystal growth. Ky15.11-S^100I^K-KQPA co-crystals grew in 2 M ammonium sulfate, 0.1 M HEPES and were cryoprotected in 15% (w/v) ethylene glycol after 6 days of crystal growth. Purified Ky15.11 Fab was concentrated and diluted to 12.5 mg/mL with NDN peptide in a 1:3 molar ratio in 20 mM sodium acetate pH 5.6 buffer. Ky15.11-NDN co-crystals grew in 0.2 M magnesium chloride, 20% (w/v) PEG8000, 0.1 M Tris pH 8.5 after micro-seeding from deformed plate-like crystals that grew in 0.2 M ammonium sulfate, 25% (w/v) PEG4000, 0.1 M sodium acetate pH 4.6 and were cryoprotected in 15% (w/v) ethylene glycol after ∼20 days of crystal growth.

Fabs of mAbs Ky315, Ky15.1, Ky311 and Ky230 underwent an additional purification step via size exclusion chromatography (Superdex 200 Increase 10/300 GL, Cytiva) prior to crystallization trials. Purified Ky315, Ky15.1 and Ky311 Fabs were then concentrated and diluted to 10 mg/mL with DND or KQPA peptide in a 1:3 molar ratio in Tris-buffered saline pH 8. Purified Ky230 was concentrated and diluted to 9.5 mg/mL with NANP_5_ in a 1:3 molar ratio in Tris-buffered saline pH 8. Ky315-DND co-crystals grew in 0.2 M magnesium formate pH 5.9, 20% (w/v) PEG3350 and were cryoprotected in 15% (w/v) ethylene glycol after 6 days of crystal growth. Ky15.1-KQPA co-crystals grew in 2.4 M ammonium sulfate, 0.1 M Tris pH 8.5 and were cryoprotected in 25% (w/v) glycerol after 5 days of crystal growth. Ky311-KQPA co-crystals grew in 0.1 M HEPES pH 7.5, 0.2 M lithium sulfate, 25% (w/v) PEG3350 and were cryoprotected in 20% (w/v) ethylene glycol after ∼35 days of crystal growth. Ky230-NANP_5_ co-crystals grew in 0.2 M di-ammonium tartrate, 20% (w/v) PEG3350 after micro-seeding from splintered rods that grew in 0.2 M ammonium acetate, 30% (w/v) PEG4000, 0.1 M tri-sodium citrate pH 5.6 and were cryoprotected in 20% (w/v) ethylene glycol after ∼40 days of crystal growth.

Ky224 Fab also underwent an additional purification step via size exclusion chromatography (Superdex 200 Increase 10/300 GL, Cytiva) prior to crystallization trials. Purified Ky224 Fab was then concentrated and diluted to 18 mg/mL with NPDP peptide in a 1:3 molar ratio in 20 mM sodium acetate, 20 mM sodium chloride. Ky224-NPDP co-crystals grew in 2 M ammonium sulfate, 0.1 M sodium acetate pH 4.6 after micro-seeding from dense, spherical microcrystals with spiky protrusions that grew in 2 M ammonium sulfate, 0.1 M sodium acetate pH 4.6 and were cryoprotected in 20% (w/v) glycerol after 25 days of crystal growth.

Data were collected at the 23-ID-B or 23-ID-D beamlines at the Advanced Photon Source (APS), the 17-ID-1 beamline at the National Synchrotron Light Source II (NSLS-II) or the CMCF-ID beamline at the Canadian Light Source (CLS) and processed and scaled using XDS.^59^ The structures were determined by molecular replacement using Phaser.^60^ Refinement of the structures was carried out using phenix.refine^61^ and iterations of refinement using Coot.^62^ Software were accessed through SBGrid.^63^ Intrachain HBs were identified by hbplus^64^; interchain HBs, BSA measurements and theoretical ΔG values were determined using the PDBePISA server,^65^ and 3/10 helices were identified by DSSP.^66^^,^^67^

### Quantification and statistical analysis

Statistical analyses were performed using GraphPad Prism version 9.4.0 (GraphPad) and R version 4.2.3. For comparison of two groups, a one- or two-tailed Mann-Whitney test or two-proportion Z-test was used, as described in the figure legends. For analysis of bite parasitemia assays, Mantel-Cox log rank tests were used with Bonferroni correction applied. For linear correlations between *in vitro* measurements and *in vivo* efficacy, simple linear regression was used. Results with *p* values less than 0.05 were considered statistically significant: ^∗^p < 0.05, ^∗∗^p < 0.01, *^∗∗∗^*p *< 0.001*.

## Data Availability

• The crystal structures reported here have been deposited to the Protein Data Bank and are publicly available as of the date of publication. Accession numbers are listed in the key resources table. • This paper does not report original code. • Any additional information required to reanalyze the data reported in this paper is available from the lead contact upon request.

## Supplemental information

Document S1. Figures S1–S3

Table S1. Isolated VH3-33 mAb sequences, ELISA AUCs, and SPR measurements, related to Figures 1 and 5

Table S2. Properties of 12 selected mAbs, related to Figure 1

Table S3. X-ray crystallography data collection and refinement data for 1210-like mAbs and variants, related to Figures 2–5

Table S4. X-ray crystallography data collection and refinement data for MGG4-like mAbs and variants, related to Figures 2–4

Table S5. X-ray crystallography data collection and refinement data for Ky230 and Ky224, related to Figures 2 and 7

Document S2. Article plus supplemental information
