# Supplementary material for: Molecular determinants of cross-reactivity and potency by VH3-33 antibodies against the Plasmodium falciparum circumsporozoite protein
Source: Cell Rep. 2023 Oct 28;42(11):113330. doi: 10.1016/j.celrep.2023.113330 (PMC10720262; doi:10.1016/j.celrep.2023.113330)
Supplement: Document S1. Figures S1–S3 [file mmc1.pdf]

Cell Reports, Volume *42*

Supplemental information

Molecular determinants of cross-reactivity and potency by VH3-33 antibodies against the *Plasmodium falciparum* circumsporozoite protein

Elaine Thai, Rajagopal Murugan, Špela Binter, Clare Burn Aschner, Katherine Prieto, Audrey Kassardjian, Anna S. Obraztsova, Ryu Won Kang, Yevel Flores-Garcia, Shamika Mathis-Torres, Kan Li, Gillian Q. Horn, Richard H.C. Huntwork, Judith M. Bolscher, Marloes H.C. de Bruijni, Robert Sauerwein, S. Moses Dennison, Georgia D. Tomaras, Fidel Zavala, Paul Kellam, Hedda Wardemann, and Jean-Philippe Julien

Figure S1

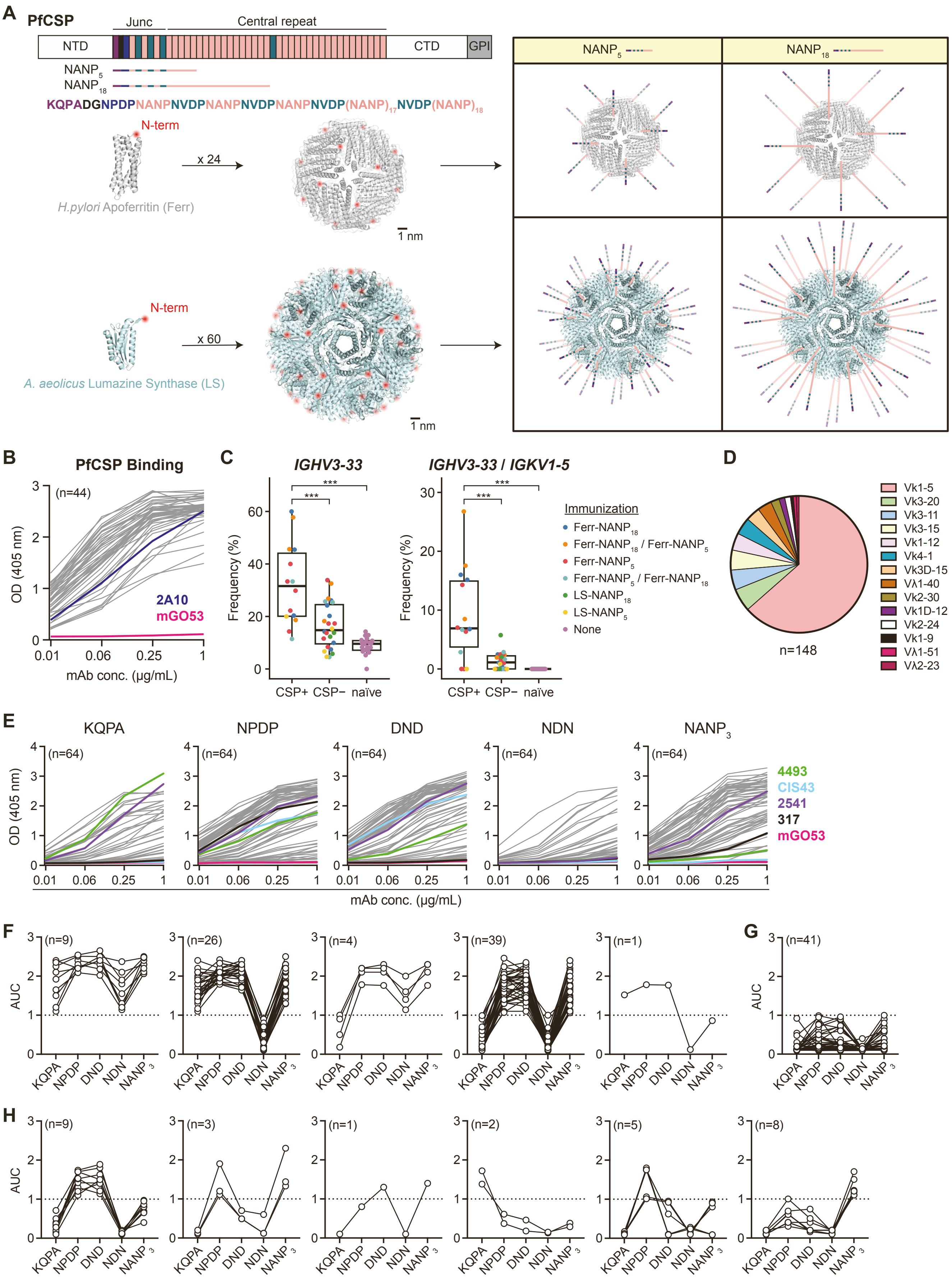

A
PfCSP
Junc
Central repeat
NTD
CTD
GPI
NANP5
NANP18
KQPADGNPDPNANPNVDPNANPNVDPNANPNVDP(NANP)17NVDP(NANP)18
N-term
x 24
H.pylori Apoferritin (Ferr)
1 nm
N-term
x 60
A. aeolicus Lumazine Synthase (LS)
1 nm
B
PfCSP Binding
(n=44)
OD (405 nm)
2A10
mGO53
mAb conc. (μg/mL)
C
IGHV3-33
IGHV3-33 / IGKV1-5
Frequency (%)
CSP+ CSP− naïve
Immunization
Ferr-NANP18
Ferr-NANP18 / Ferr-NANP5
Ferr-NANP5
Ferr-NANP5 / Ferr-NANP18
LS-NANP18
LS-NANP5
None
D
n=148
Vκ1-5
Vκ3-20
Vκ3-11
Vκ3-15
Vκ1-12
Vκ4-1
Vκ3D-15
Vλ1-40
Vκ2-30
Vκ1D-12
Vκ2-24
Vκ1-9
Vλ1-51
Vλ2-23
E
KQPA
NPDP
DND
NDN
NANP3
(n=64)
OD (405 nm)
4493
CIS43
2541
317
mGO53
mAb conc. (μg/mL)
F
(n=9)
(n=26)
(n=4)
(n=39)
(n=1)
AUC
G
(n=41)
H
(n=9)
(n=3)
(n=1)
(n=2)
(n=5)
(n=8)
KQPA NPDP DND NDN NANP3

Figure S1. Immunogen design and down-selection of elicited VH3-33 mAbs, related to Figure 1. (A) Four immunogens containing PfCSP junction and central repeat motifs were designed using the *Helicobacter pylori* apoferritin (Ferr) and *Aquifex aeolicus* lumazine synthase (LS) nanocage backbones. A schematic of PfCSP from strain NF54 is shown with domains labelled as follows: N-terminal domain (NTD), junctional region (Junc), C-terminal domain (CTD), GPI anchor (GPI). The junctional and central repeat regions are coloured according to the sequence displayed below the schematic. Designed immunogens display the entire PfCSP junction followed by 5 ($NANP_5$) or 18 ($NANP_{18}$) consecutive NANP repeats. Ferr (PDB ID: 3BVE) and LS (PDB ID: 1HQK) monomers and fully assembled nanocages are shown [1,2]. PfCSP-derived segments were fused to the N-termini of Ferr and LS monomers, highlighted in red. Schematics representing the resulting nanocage immunogens are shown to the right. (**B and E)** Representative ELISA binding curves of 44 mAbs to PfCSP (B) and 64 mAbs to the indicated peptides (E) at four different mAb dilutions. Curves corresponding to positive (mAbs 2A10, 2541, 317, 4493 and CIS43) and negative (mAb mGO53) controls are coloured, as indicated by the legend to the right. **(C)** Frequency of antibodies encoded by *IGHV3-33* (left) and *IGHV3-33*/*IGKV1-5* (right) in CSP+ and CSP- lymph node B cells upon immunizing mice from the Kymouse™ platform with the indicated immunogens compared to naïve B cells from unimmunized mice. Dots indicate individual mice. Statistical significance determined by one-tailed Mann-Whitney test: ***P < 0.001. **(D)** Distribution of light chain Ig gene segments of isolated PfCSP-reactive VH3-33 mAbs. **(F-H)** ELISA binding profiles of cross-reactive mAbs that exhibited binding to at least three of the five indicated peptides (F), mAbs with weak binding to each of the five peptides (G) or mAbs that bound only one or two of the peptides (H). Dotted line indicates binding threshold (AUC > 1). Data represent mean of three independent experiments. **(B and D-H)** n indicates the number of sample mAbs.

Figure S2

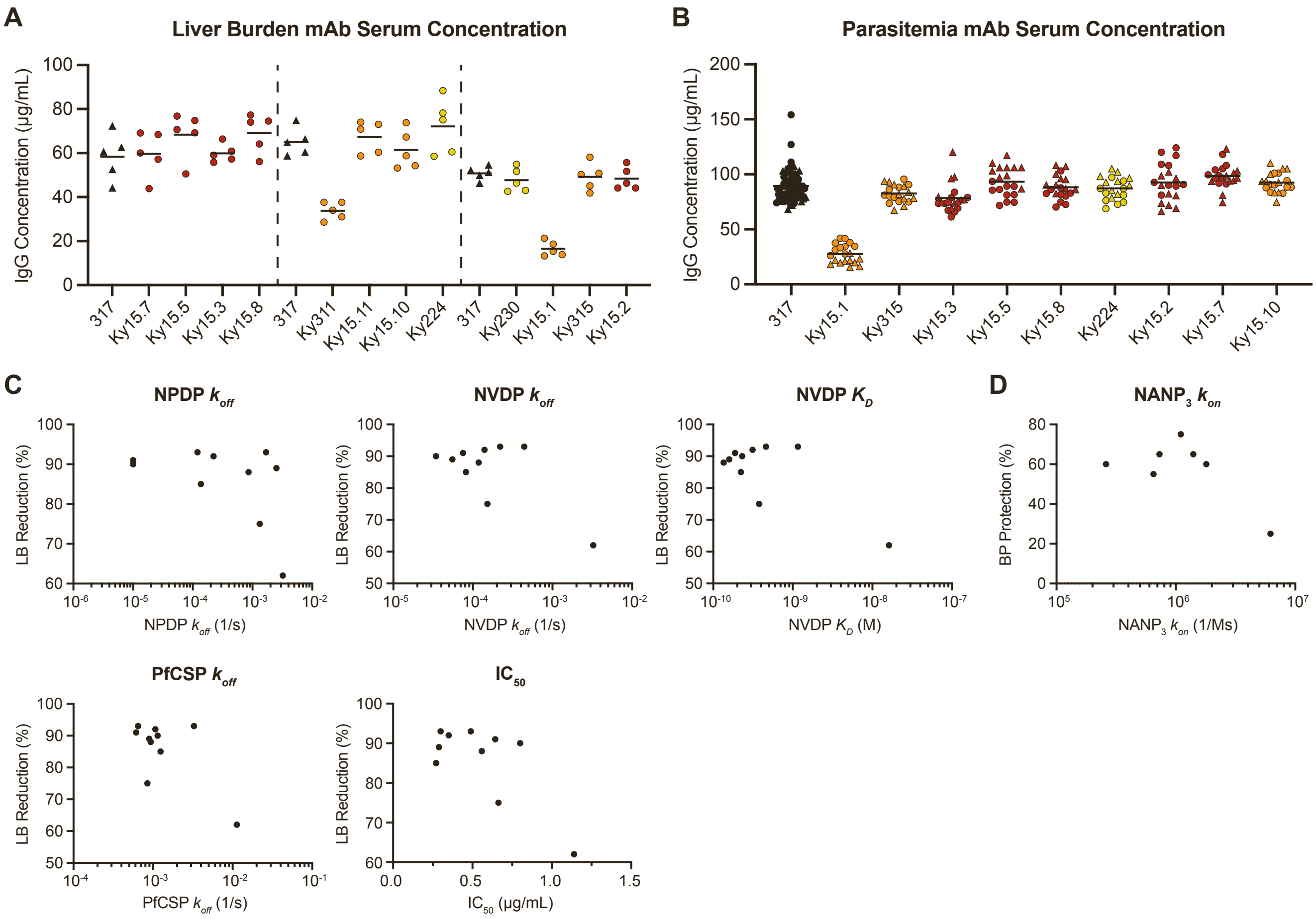

Figure S2. Details of high-affinity cross-reactive VH3-33 mAb broad parasite inhibitory capacity, related to Figure 1. (A) Antibody serum concentration one hour prior to time of challenge in the liver burden assay. mAb 317 was used as a positive control and is plotted as black triangular symbols. Symbols represent individual mice (n=5) and black dashed lines separate independent experiments. **(B)** Antibody serum concentration one hour prior to time of challenge in the parasitemia experiment. mAb 317 was used as a positive control and is plotted as black symbols. Symbols represent individual mice (n=20 for mAb samples and n=70 for mAb 317) with circular and triangular symbols representing independent replicate experiments for each mAb sample and the corresponding mAb 317 measurements. **(A and B)** mAb symbols are coloured based on cross-reactivity, as in Figure 1C-H. Black lines indicate arithmetic mean. **(C and D)** *In vitro* measurements found to have a significantly non-zero slope through simple linear regression analysis with liver burden (LB) % reduction (C) or bite-parasitemia (BP) % protection (D). Simple linear regression analyses were performed in GraphPad Prism. mAbs that yielded mean serum antibody titers < 40 μg/mL or < 50 μg/mL one hour prior to liver burden or parasitemia challenge, respectively, are excluded. Symbols represent the arithmetic mean of corresponding measurements.

Figure S3

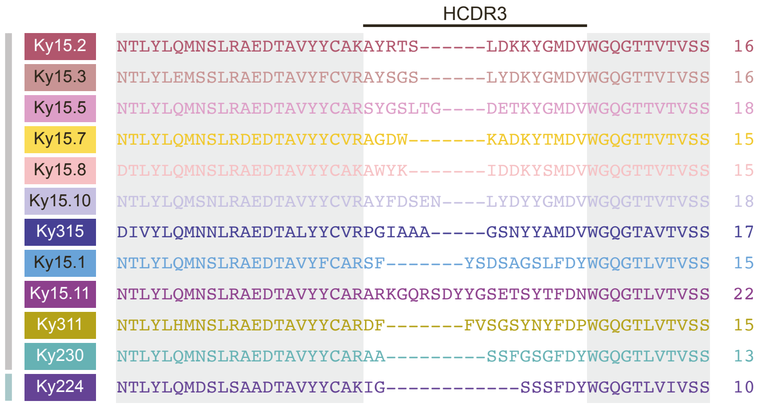

Figure S3. HCDR3 sequence alignment for the 12 selected VH3-33 mAbs, related to Figure 3. Sequences are coloured by mAb with framework residues shaded in grey and HCDR3 residues as labelled. HCDR3 amino acid length is indicated on the right side for each mAb. Grey and pale cyan bars on the left side correspond to the C-core conformation induced by each mAb, as described in Figure 3. Sequence alignment was conducted using Clustal Omega [3].

SUPPLEMENTAL REFERENCES

1. Cho, K.J., Shin, H.J., Lee, J.-H., Kim, K.-J., Park, S.S., Lee, Y., Lee, C., Park, S.S., and Kim, K.H. (2009). The crystal structure of ferritin from *Helicobacter pylori* reveals unusual conformational changes for iron uptake. J Mol Biol *390*, 83–98. 10.1016/j.jmb.2009.04.078.
2. Zhang, X., Meining, W., Fischer, M., Bacher, A., and Ladenstein, R. (2001). X-ray structure analysis and crystallographic refinement of lumazine synthase from the hyperthermophile *Aquifex aeolicus* at 1.6 Å resolution: Determinants of thermostability revealed from structural comparisons. J Mol Biol *306*, 1099–1114. 10.1006/jmbi.2000.4435.
3. Sievers, F., Wilm, A., Dineen, D., Gibson, T.J., Karplus, K., Li, W., Lopez, R., McWilliam, H., Remmert, M., Söding, J., et al. (2011). Fast, scalable generation of high-quality protein multiple sequence alignments using Clustal Omega. Mol Syst Biol *7*, 539. 10.1038/MSB.2011.75.
